# Perinatal lead (Pb) exposure and alterations to amyloid beta and metabolic profiles in the brain

**DOI:** 10.1002/alz.71791

**Published:** 2026-08-30

**Authors:** Rachel K. Morgan, Evelyn K. Matei, Junru Pan, Tomoko Ishikawa, Katelyn M. Polemi, Ingrid Bergin, Sean P. Ferris, Hao Wang, Dana C. Dolinoy, Kelly M. Bakulski, Justin A. Colacino

**Affiliations:** ^1^ Department of Environmental Health Sciences School of Public Health, University of Michigan Ann Arbor Michigan USA; ^2^ School of Medicine University of Michigan Ann Arbor Michigan USA; ^3^ Department of Nutritional Sciences School of Public Health, University of Michigan Ann Arbor Michigan USA; ^4^ Unit for Laboratory Animal Management University of Michigan Ann Arbor Michigan USA; ^5^ Department of Epidemiology School of Public Health, University of Michigan Ann Arbor Michigan USA; ^6^ Program in the Environment College of Literature, Sciences, and the Arts and School of Sustainability, University of Michigan Ann Arbor Michigan USA

**Keywords:** amyloid, lead, metabolomics, neurodegeneration, neurotoxicology, pathology

## Abstract

**INTRODUCTION:**

Developmental neurotoxicant exposures (e.g., lead [Pb]) are suspected contributors to dementia. Dementia is characterized by amyloid beta (Aβ) plaques and dysregulated metabolism. To better understand the relationship between environment and dementia risk, we assessed the impact of perinatal Pb exposure on dementia‐related outcomes in the murine brain.

**METHODS:**

Female mice were exposed to 32 ppm Pb or control water during gestation and lactation. Offspring were aged out to 3 weeks or 18 months. We measured Aβ in the 18‐month cortex and performed untargeted metabolomics in plasma and cortex at 3 weeks and 18 months of age.

**RESULTS:**

Diffuse Aβ and Aβ‐positive cells were significantly elevated with Pb exposure. Pb‐associated metabolites were enriched for those implicated in oxidative stress, inflammation, and lipid metabolism.

**DISCUSSION:**

Pb was associated with molecular and metabolic features of dementia. Important biochemical classes provide insights into possible mechanisms by which developmental Pb exposure may contribute to dementia risk.

## Highlights

- This study assessed the effects of developmental lead (Pb) exposure on brain pathology and metabolic profiles in adult murine brain.
- Diffuse amyloid beta (Aβ) and Aβ‐positive cells were significantly elevated with Pb exposure.
- Pb‐associated metabolites were enriched for those implicated in oxidative stress, inflammation, and lipid metabolism.
- Developmental exposure to human‐relevant levels of Pb produces persistent, late‐life Alzheimer's disease–related neuropathologic and metabolic alterations in the cortex.
- Our study provides experimental evidence that an early‐life environmental toxicant can durably reprogram trajectories of brain aging and vulnerability to neurodegenerative disease.

## 1 BACKGROUND

Alzheimer's disease (AD), characterized by progressive cognitive decline and neurodegeneration as well as pathological molecular features including amyloid beta (Aβ) accumulation, is a growing public health challenge.^1^ In 2024, nearly 7 million people in the United States were living with AD,^1^ and prevalence is expected to double by 2060,^2^ given a rapidly aging population.^3^ While genetics and familial risk factors play an important role in AD,^4^ modifiable risk factors warrant further exploration as it is estimated they account for 40% of disease risk.^1^ Significant knowledge gaps remain regarding the impact environmental toxicants have on AD particularly that of the metal lead (Pb), which has known neurotoxic effects and persistent exposures in the United States.^5^

AD‐relevant molecular and metabolic changes accumulate over decades, often preceding clinical symptoms by many years.^6^ This long preclinical window suggests that late‐life dementia risk may be shaped not only by proximal adult exposures and genetics, but also by early‐life environments that “program” brain development and aging trajectories, consistent with the developmental origins of health and disease (DOHaD) paradigm.^7^ However, testing early‐life determinants of AD in humans is challenging because exposure assessment is often retrospective, and many epidemiologic studies remain cross‐sectional or lack prospective measurements spanning from gestation into later life.^8^ Consequently, there is a critical need for animal models, yet relatively few developmental toxicology studies extend follow‐up into mid‐to‐late adulthood (≥ 17 months in mice), when AD‐related and age‐dependent molecular changes and metabolic dysregulation are most likely to manifest.^9^

Pb exposure is a significant public health concern, and developmental Pb exposure is of particular interest in a DOHaD framework because the fetal and early post‐natal brain undergoes rapid neurogenesis, synaptogenesis, and metabolic programming that may set long‐term vulnerability. Roughly 1.8 million children under the age of 5 in the United States have blood Pb levels > 3.5 µg/dL,^10^ and 50% of the US population has been exposed to high levels of Pb (blood Pb > 17.5 µg/dL) during childhood.^11^ Indeed, Pb exposure in adulthood is associated with more than two times greater risk for incident AD over 30 years of follow‐up.^12^ Neurotoxicant exposures during development may result in mature neurological systems that are more susceptible to AD progression later in life^13^, ^14^ through increased oxidative stress, suboptimal neurogenesis, and neuroinflammation.^15^ Thus, a potential mechanistic link between Pb exposure and AD susceptibility may be through alterations in metabolism. For example, *post mortem* brain tissues from participants with AD have altered cholesterol metabolism, oxidative stress, neuroinflammation, and bioenergetics, relative to those with normal cognition,^16^, ^17^ pathways which may also be labile to exposures to heavy metals like Pb.

Understanding the modifiable risk factors for AD and their mechanisms of action is critical to developing effective disease prevention, early detection, and treatment strategies. Here, we assessed the effects of developmental Pb exposure on brain molecular features related to AD and metabolic profiles in a mouse model (C57BL/6J). We examined neuronal necrosis and mineralization, as well as the presence of Aβ in the cortex at 18 months of age after perinatal exposure to Pb, > 17 months after the cessation of exposure. To characterize alterations in metabolic pathways driven by developmental Pb exposure, we performed untargeted metabolomic profiling of the cortex at both an early (3 weeks of age) and a late (18 months of age) time point. To determine the utility of these metabolic alterations in circulating tissue as effective biomarkers of Pb exposure and disease risk, we also performed metabolomic profiling of plasma from the same mice. We hypothesized that perinatal Pb exposure is associated with increased Aβ in aged mice and perturbed metabolomic pathways associated with AD risk.

## 2 METHODS

### 2.1 Exposure paradigm, tissue collection, and processing

Virgin C57BL/6J female mice (6–8 weeks old, Jackson Laboratories, Cat. #000664) were randomly assigned to control or Pb‐acetate water 2 weeks prior to mating with virgin C57BL/6J males (8–10 weeks old). Pb exposure was conducted ad libitum via distilled drinking water mixed with Pb‐acetate at a concentration of 32 ppm to model human‐relevant perinatal exposure. We have previously demonstrated that 32 ppm Pb delivered via drinking water results in maternal blood Pb levels of 16 to 60 µg/dL (mean = 32.1 µg/dL Pb) in mice^18^ and represents a dose that, when detected in pregnant humans, would initiate treatment for a high‐risk pregnancy due to Pb toxicity.^19^ All mice were maintained on a phytoestrogen‐free modified AIN‐93 G diet (Td.95092, 7% corn oil diet, Envigo) while housed in polycarbonate‐free cages. Exposure to Pb continued through F0 generation mating, gestation, and lactation until F1 offspring weaning at post‐natal day 21 (PND21) when pups were switched to Pb‐free drinking water. F1 offspring were maintained until either PND21 or 18 months of age.

**RESEARCH IN CONTEXT**

1. **Systematic review**: Alzheimer's disease (AD) is a public health challenge that is driven, in part, by modifiable risk factors, including environmental exposures. AD has a long preclinical period, suggesting that AD risk may be shaped by early environments that “program” brain development and aging. Here, we assessed the effects of developmental lead (Pb) exposure on brain pathology and metabolic profiles in adult mice to test this theory.
2. **Interpretation**: Pb exposure was associated with elevated amyloid beta in the brain, a feature of AD, and perturbed metabolism related to AD, including markers of oxidative stress, inflammation, and dysregulated lipid metabolism.
3. **Future directions**: Consistent Pb‐associated metabolites in plasma and cortex provide avenues for future epidemiological biomarker work. Metabolic changes associated with related health conditions to AD, such as preeclampsia, warrant additional toxicological investigations related to comorbidities. Continued work in AD‐relevant mouse models would help solidify the preliminary relationships established here.

**FIGURE 1:**
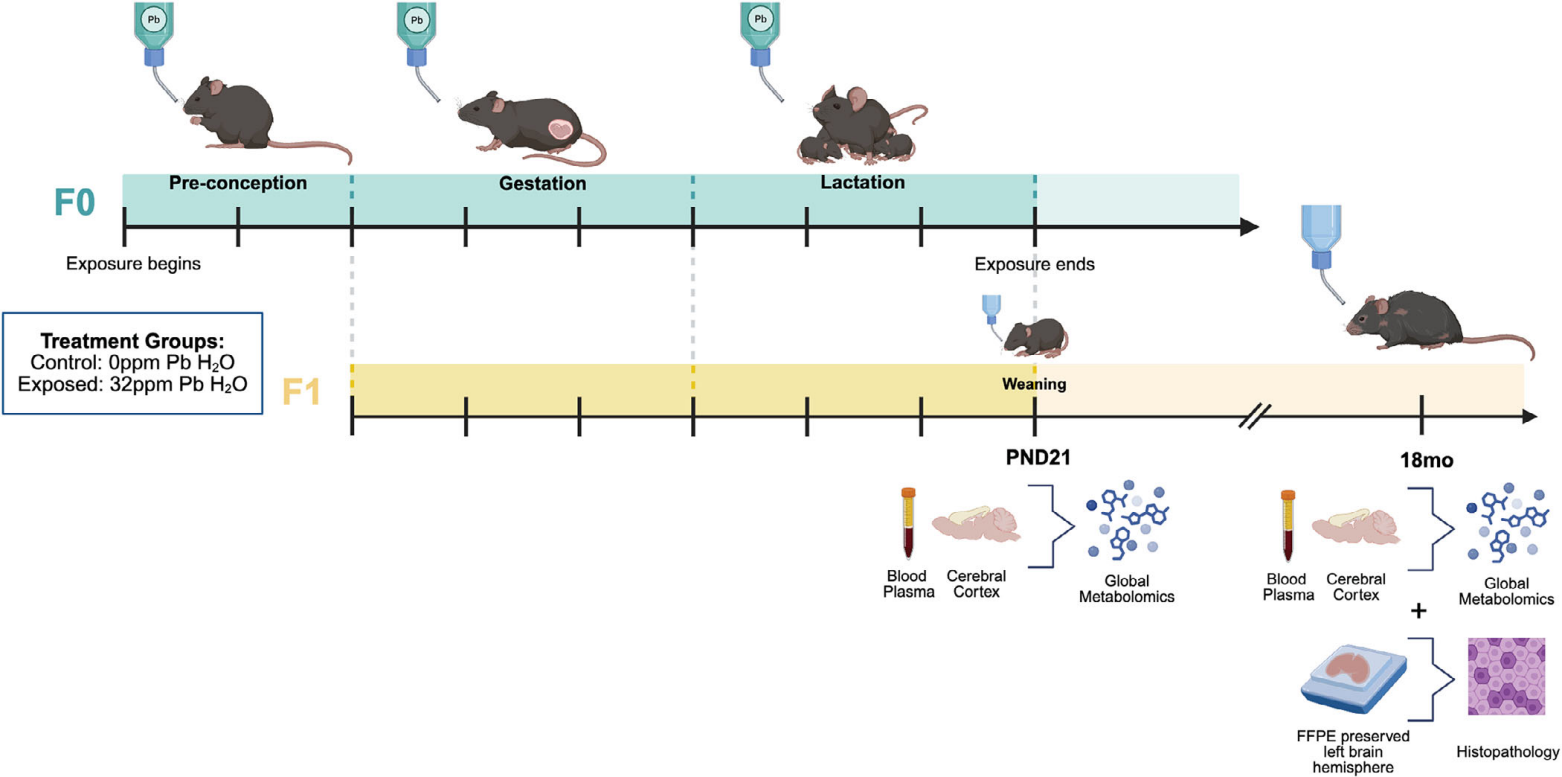
Experimental design. Female C57BL/6J mice (6–8 weeks of age) were randomly assigned to exposure (32 ppm lead [Pb] in drinking water) or control (0 ppm Pb in drinking water) groups 2 weeks prior to mating with male C57BL/6 mice (8–10 weeks of age). Exposure via drinking water continued throughout mating, gestation, and lactation, and ceased when offspring (F1) were weaned at post‐natal day 21 (PND21). F1 offspring were either sacrificed for tissue collection at PND21 or aged out to 18 months of age, at which point cerebral cortex and blood tissues were collected for untargeted metabolomics. Cortex tissue was flash frozen and cryopulverized and blood plasma was separated from whole blood samples via centrifugation. Samples were sent to Metabolon for non‐targeted analysis using their Global Discovery Panel via ultra‐performance liquid chromatography (UPLC)‐mass spectrometry (MS). Additionally, cerebral cortex was collected from 18‐month‐old animals and formalin fixed and paraffin embedded (FFPE). Samples were processed by the University of Michigan Pathology Core via hematoxylin and eosin (H&E) and Luxol fast blue (LFB) to evaluate necrosis, focal mineralizations, and immunostained for amyloid beta.

Tissue collections and processing followed previously published protocols with no necessary deviations.^20^ Immediately after mouse euthanasia with CO_2_ asphyxiation, blood was collected through cardiac puncture and perfusion performed with 1X phosphate‐buffered saline (Thermo, Cat. # 10010023). Blood was collected in ethylenediaminetetraacetic acid‐coated tubes (Fisher, Cat. #365974) and plasma isolated via centrifugation for 10 minutes at 500 g at 4°C. Blood plasma was stored at −80°C until metabolomic profiling. The brain was removed, and the olfactory bulbs and brain stem were discarded. The whole brain was sectioned sagitally into the left and right hemispheres. The left hemisphere was preserved in 4% paraformaldehyde (18‐month time point only), and the right hemisphere was further microdissected to isolate the cerebral cortex (in PND21 and 18‐month‐old animals), which was immediately flash frozen in liquid nitrogen. Frozen cerebral cortex tissue was cryopulverized at the University of Michigan Epigenomics Core for metabolomic analyses. Animal euthanasia and tissue collections were standardized to between 9:00 am and 12:00 pm and collection order was randomized daily. The PND21 time point included 8 control and 8 Pb exposed males, and 11 control and 11 Pb exposed female mice (*n_pnd21_* = 27). The 18‐month time point included 9 control and 10 Pb exposed males, and 11 control and 10 Pb exposed females (*n_18‐mo_* = 39). A description of exposure methods is provided in Figure 1.

### 2.2 Brain histopathology and immunohistochemical staining

All histopathological experiments were conducted in collaboration with the University of Michigan Pathology Core. Four percent paraformaldehyde‐preserved left brain hemispheres from 18‐month‐old mice were formalin fixed and paraffin embedded (FFPE). This work included 10 female exposed, 11 female control, 10 male exposed, and 9 male control samples. Brain tissue was further trimmed in the coronal plane post‐fixation into 4 mm thick standardized section levels, allowing for an overview of cerebral cortex, anterior commissure, basal ganglia, corpus callosum, thalamic nuclei, hippocampus, cerebral peduncles, midbrain, pons, cerebellum, and cerebellar pyramids (i.e., section levels 2, 3, 5 in Society for Toxicologic Pathology best practices guidelines for sampling the rodent brain^21^). Sections were cassetted and processed to paraffin on an automated histology processor (Tissue Tek VIP, Sakura). Slides were embedded and sectioned at 4 µm thickness onto glass slides for staining with hematoxylin and eosin (H&E) or Luxol fast blue (LFB). Slides were evaluated by a board‐certified veterinary pathologist (I.B.) using a BX45 Olympus light microscope. Slides were scanned on a digital slide scanner (Leica Aperio AT2, Leica Biosystems) at a resolution of 0.50 µm/pixel (20x objective equivalent). Representative images were taken from the digital slides using manufacturer‐provided software (Leica Aperio ImageScope v 12.4.6, Leica Biosystems). Composite images were assembled in Adobe Photoshop CC (v 19.0, Adobe Systems Inc.). Photo processing was limited to global adjustments to image size, white balance, brightness, or contrast that did not materially alter the image's interpretation. A summary of initial histological methods is provided in the **Supplementary Document** in supporting information.

Additional slides underwent deparaffinization and heat‐induced epitope retrieval (HIER) in a pressure chamber (Decloaking Chamber, Biocare Medical) and immunochemical staining was performed on an automated stainer (ONCORE Pro X, Biocare Medical), including blocking of endogenous peroxidases, primary antibody (ab201060, Abcam) incubation, and polymer‐based detection. The primary antibody (rabbit monoclonal, clone mOC64) targets monomeric, oligomeric, and fibrillar forms of Aβ1–42 peptide. An archival brain tissue sample from a murine AD model with histologically evident Aβ plaques was used for initial antibody titration and as a positive control. Conditions and reagents used in primary antibody incubation and detection are provided in the **Supplementary Document**. Diaminobenzidine (DAB) was applied for 5 minutes as the chromogen (IPK5010G80, Biocare Medical). After immunohistostaining, samples were counterstained with hematoxylin for 5 minutes, dehydrated and cleared through graded alcohols and xylene, and cover‐slipped. Negative reagent controls followed the same procedure with naïve serum (Universal Negative, IP498G20, Biocare Medical) in place of primary antibody.

### 2.3 Immunohistology analysis

Descriptive and quantitative analysis was performed by a board‐certified veterinary pathologist (I.B.) blinded to the experimental groups. Histology slides were digitized at a resolution of 0.27 µm^2^/pixel on a digital slide scanner (Slideview VS200, Evident Scientific). Digital slide files were imported into an open‐source image analysis program, QuPath^22^ (v0.6.0). Image analysis was performed on a workstation equipped with a 12^th^‐generation Intel Core Processor (i7‐14700K, 33 MB cache, 20 cores, 28 threads, up to 5.6 GHz Turbo) with 128 GB of installed memory and an additional 8GB installed GPU (NVIDIA, GeForce RTX 4060). Representative image overlays were generated within QuPath and exported as tiff files. Intensity of DAB staining was first assessed across the entire tissue area using a pixel classifier for tissue detection and algorithms for stain intensity assessment within QuPath. Color deconvolution was first performed by adjusting RGB stain parameters based on small annotations representing single‐stained areas for hematoxylin and DAB. The entire brain sections were automatically selected using a pixel classifier thresholded based on the average intensity across all color channels. Annotations were reviewed by the pathologist and manually exited to exclude artifacts and include any true tissue regions erroneously omitted by the classifier. Average and standard deviation for DAB intensity were measured across the whole section.

Cells were segmented within the tissue annotation using a previously trained cell segmentation model (InstanSeg) available within QuPath as an extension (https://arxiv.org/abs/2408.15954). Because axonal and dendritic projections of neuronal cells are not definable at the resolution of light microscopy, only nuclei and cell bodies were identified for segmentation. Cell bodies positive for DAB were identified using a cell classifier plugin within QuPath. Thresholds for determining positive cells were defined empirically compared to the negative control, non‐staining areas, and lightly staining areas. Thresholds were applied equally across all tissue sections. High‐intensity staining seen in capillaries and foci of mineralization were identified using a pixel classifier within QuPath. The threshold‐defining high‐intensity staining was determined empirically by visual assessment of the digital overlays and was applied equally across all tissue sections. Representative field‐of‐view images were taken from the digital slide files as tiff files and composite panels were assembled in Adobe Photoshop CC (v19.0). Photo processing was confined to global adjustments of image size, white balance, brightness, or contrast that did not materially alter the interpretation of the image.

### 2.4 Sample processing for untargeted metabolomic profiling with metabolon

Frozen cortex and blood plasma samples were submitted to Metabolon for untargeted metabolomic profiling via their Global Discovery Panel. Sample submission requirements included a minimum tissue weight (cerebral cortex) of 30 mg and a minimum liquid volume (blood plasma) of 150 µL. After screening for these metrics, our final sample size was adjusted to PND21 cerebral cortex: 8 control male, 11 Pb exposed male, 7 control female, and 11 Pb exposed female; PND21 blood plasma: 8 control male, 10 Pb exposed male, 8 control female, and 9 Pb exposed female; 18‐month cerebral cortex: 9 control male, 10 Pb exposed male, 10 control female, and 10 Pb exposed female; 18‐month blood plasma: 9 control male, 7 Pb exposed male, 10 control female, and 9 Pb exposed female samples, for a total sample size of *N_cerebral cortex_* = 76 and *N_blood plasma_* = 70. A summary of all exposure groups, summarized by age, sex, and tissue type, is provided in Table S1 in supporting information.

All samples were shipped on dry ice to Metabolon and stored at −80°C until untargeted metabolomic profiling via their Global Discovery Panel, which can identify as many as 5400 metabolites within biological samples. A full description of methods is in the **Supplementary Document**. Briefly, samples were prepared using the automated MicroLab STAR system (Hamilton Company), and several recovery standards were added prior to the first extraction step for quality control (QC) processing. Protein precipitation was achieved via methanol addition and vigorous shaking for 2 minutes (Glen Mills GeoGrinder 2000) and centrifugation. Extract was divided into multiple fractions: two for analysis by two separate reverse phase (RP)/ultra‐performance liquid chromatography (UPLC)‐mass spectrometry (MS) methods with positive ion mode electrospray ionization (ESI) and RP/UPLC‐MS with negative ion mode ESI, one for analysis by hydrophilic interaction chromatography (HILIC)/UPLC‐MS/MS with negative ion mode ESI, and the remaining fractions were reserved. Samples were placed briefly on a TurboVap (Zymark) to remove organic solvent and stored overnight under nitrogen before analysis.

### 2.5 Ultra‐high‐performance liquid chromatography‐tandem mass spectroscopy

Dried sample extracts were reconstituted in solvents compatible with each of the four methods, each containing a series of standards at fixed concentrations to ensure injection and chromatographic consistency. One aliquot was analyzed using acidic positive ion conditions, chromatographically optimized for hydrophilic compounds (PosEarly), and another using acidic positive ion conditions chromatographically optimized for hydrophobic compounds (PosLate). Additionally, an aliquot was analyzed using basic negative ion conditions (Neg) with extracts eluted from the column using methanol and water and 6.5 mM ammonium bicarbonate (pH = 8). A final aliquot was analyzed via negative ionization after elution from a HILIC column using a gradient consisting of water and acetonitrile with 10 mM ammonium formate (pH = 10.8). The MS analysis alternated between MS and data‐dependent MS scans using dynamic exclusion and the scan range covered 70 to 1000 m/z.

### 2.6 Data extraction and compound identification and quantification

Raw data were extracted, peak identified, and QC processed using a combination of Metabolon‐developed software services. Compounds were identified compared to library entries of purified standards or recurrent unknown entities. Metabolon maintains a library based on authenticated standards that contains the retention time/index (RI), mass to charge ratio (m/z), and fragmentation data on all molecules present in the library. Biochemical identification is based on three criteria: (1) RI within a narrow window of the proposed identification, (2) accurate mass match to the library ± 10 ppm, and (3) the MS/MS forward and reverse scores between the experimental data and authentic standards. The MS/MS scores are based on a comparison of the ions present in the experimental spectrum to the ions present in the library spectrum. More than 5400 commercially available purified or in‐house synthesized standard compounds have been acquired and analyzed on all platforms for determination of their analytical characteristics. An additional 7000 mass spectral entries have been created for structurally unnamed biochemicals, which have been identified by virtue of their recurrent nature (both chromatographic and mass spectral). Peaks were quantified using area under the curve.

### 2.7 Statistical analyses and data visualization

Histopathological data were evaluated by either Fisher exact tests to compare the presence or absence of mineralization foci between samples groups, or two‐sided *t* tests to compare the amount of Aβ staining intensity as well as the number of Aβ‐positive cells within the brain hemispheres. For both statistical tests, significance was accepted at *P* < 0.05.

Metabolomic data were reported as fold changes (FCs) in biochemical concentration in the Pb‐exposed samples relative to the control, with FCs > 1 indicating higher concentrations in the Pb‐exposed tissues and FCs < 1 indicating lower concentrations in the Pb‐exposed tissues. All comparisons are reported as differences in biochemical concentration in the Pb‐exposed samples relative to control for a given sample set. Metabolomic data were evaluated using two‐way analysis of variance (ANOVA) to assess the main effect of each factor (sex, exposure group) and the interaction. Significance was accepted at *P* < 0.05. *q* values are presented alongside *P* values as a method of describing the false discovery rate.

We performed enrichment analyses using MetaboAnalyst, an online platform for evaluating metabolomic data.^23^ Sets of biochemicals significantly associated with Pb were input into the Pathway Analysis tool. Enrichment was tested using a hypergeometric test to evaluate enrichment in documented pathways within the Mus musculus (Kyoto Encyclopedia of Genes and Genomes) pathway library. We also evaluated Pb‐associated biochemical enrichment in characterized health outcomes using the Enrichment Analysis tool and metabolite disease library for human blood samples. All resulting enrichment data were imported into R (v.4.3.3). Data visualization was achieved using the pheatmap and ggplot packages.

## 3 RESULTS

### 3.1 Perinatal Pb exposure is not associated with gross histological alterations in the aged brain

We first evaluated H&E and LFB stained slides of the brain hemispheres from 18‐month‐old Pb‐exposed and control mice for cellular necrosis and cell number. There was no evidence of neuronal, endothelial cell, or vascular necrosis. There were no overtly abnormal decreases in neural number and/or density, and no differences were readily detectable in myelin content or distribution. Glial cell number and distribution appeared normal. Subtle differences in neuronal or glial cell number would require quantitative analysis, which was not performed. Representative histological images are provided in Figure S1 in supporting information.

Focal mineralization within the thalamus was noted in 9 of the 41 samples evaluated (Table S1). This consisted of focal small globular or linear mineralized aggregates within the thalamic parenchyma. Some appeared to arise from capillaries. Mineralization in other areas was not present and there were no other lesions accompanying these foci. Small foci of mineralization are sometimes seen in the brain of mice as a background lesion of unknown cause.^24^, ^25^ There was no statistical difference in mineralization foci presence between Pb exposed and control groups, using either H&E or LFB images for either males (H&E *P* value = 0.62, LFB *P* value = 1) or females (H&E *P* value = 1, LFB *P* value = 1).

Dark neuron artifact and sporadic vacuolation within the cerebellar arbor vitae were present in some samples. These are common artifactual changes in rodent brain histology.^26^ No areas of true neuronal necrosis or secondary changes like glial nodules or gliosis accompanied these findings, supporting that they were artifactual. One sample has a few very small foci of neuronal heterotopia within the molecular layer of the cerebellar paraflocculus (Table S2 in supporting information). Heterotopia is the presence of normal cells in a non‐normal location and, in the brain, usually represents a neuronal migration anomaly during development. Small foci of heterotopia can be background findings in mice and are typically of no clinical significance unless pronounced or associated with other anomalies.^26^ Overall, there was no strong histological evidence of Pb‐associated damage in these samples.

### 3.2 Perinatal Pb exposure is associated with increased diffuse Aβ in the aged brain

**FIGURE 2:**
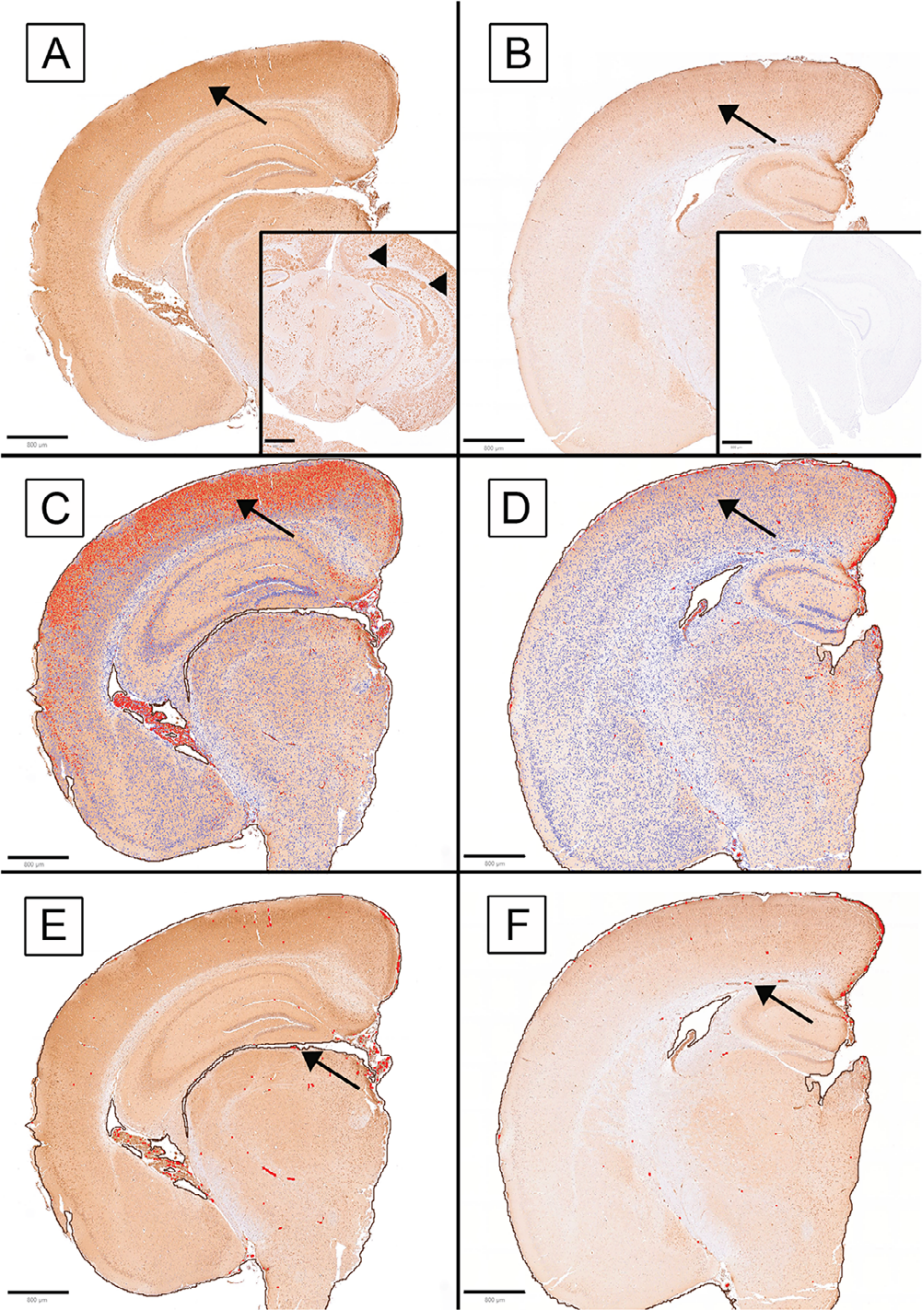
Representative low‐magnification images of amyloid beta immunostained cerebral cortex. Exposed Female Sample 1 showed diffuse moderate‐ to high‐intensity staining in gray matter (A) and Control Female Sample 2 showed diffuse low‐ to moderate‐intensity staining in the same areas (B). A positive control brain section from a murine Alzheimer's disease model (A, inset) and negative control (B, inset) are also shown. Digital overlays showing positive (red) and negative (blue) cells are shown in (C) and (D) and digital overlays of the highest intensity pixel areas are shown in (E) and (F). Bars 1 mm.

**FIGURE 3:**
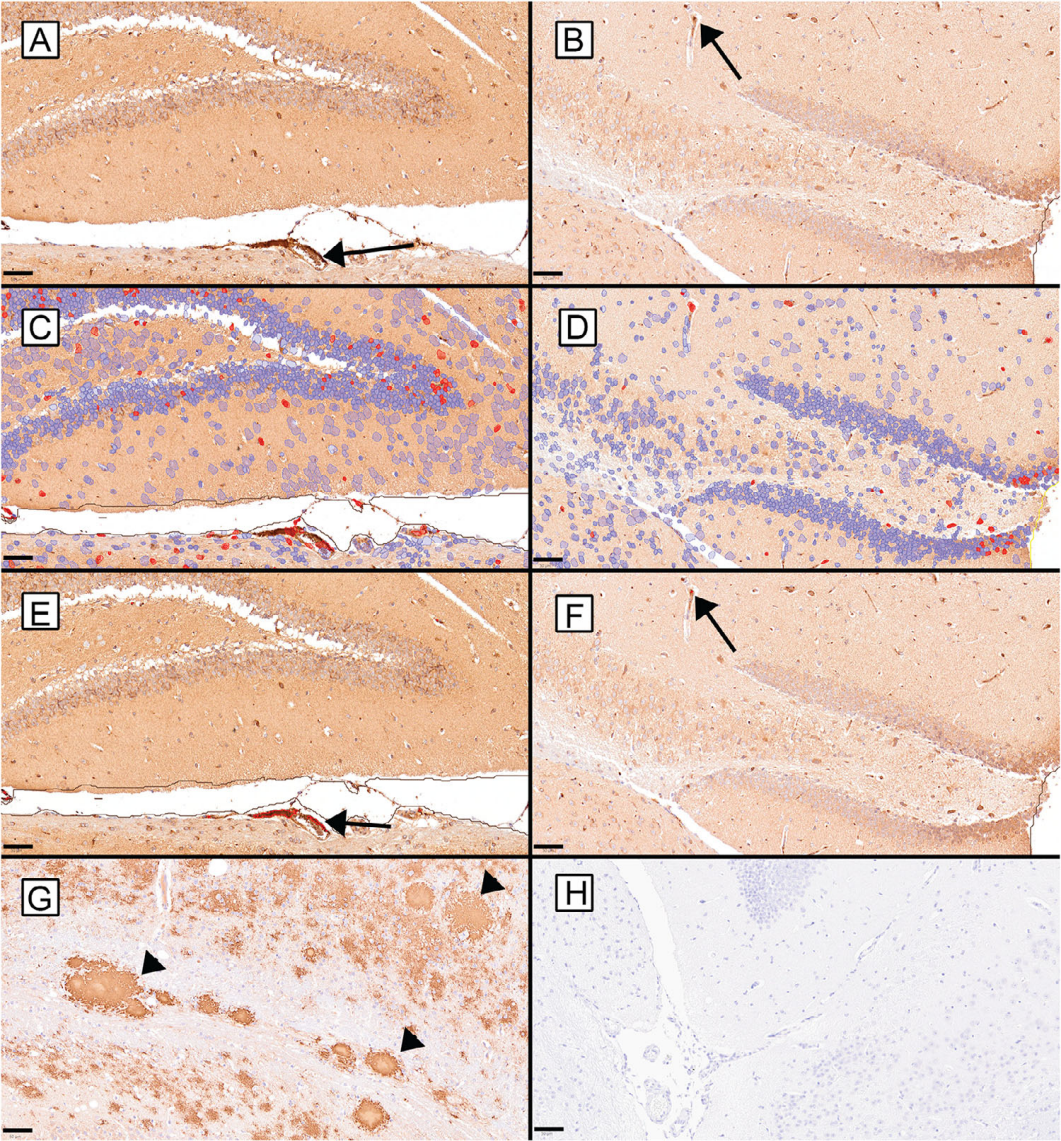
High‐magnification representative images of amyloid beta (Aβ) immunostained cerebral cortex. Panels include Female Exposed Sample 1 (A,C,E), Female Control Sample 2 (B,D,F), a positive control brain section from a murine Alzheimer's disease model (G), and a negative control (H). Sample 1 showed diffuse moderate‐ to high‐intensity staining in gray matter (A) and Sample 2 showed diffuse low‐ to moderate‐intensity staining in the same areas (B). Both samples showed highest intensity staining in vascular walls (arrows, A, B). Digital overlays of positive (red) and negative (blue) cells are shown in (C) and (D) and digital overlays of the highest intensity pixel areas (arrows) are shown in (E) and (F). The positive control (G) shows numerous extracellular Aβ plaques (arrowheads) and also Aβ staining in vascular walls (arrow). Negative control shows no staining (H). Bars 50 µm.

**FIGURE 4:**
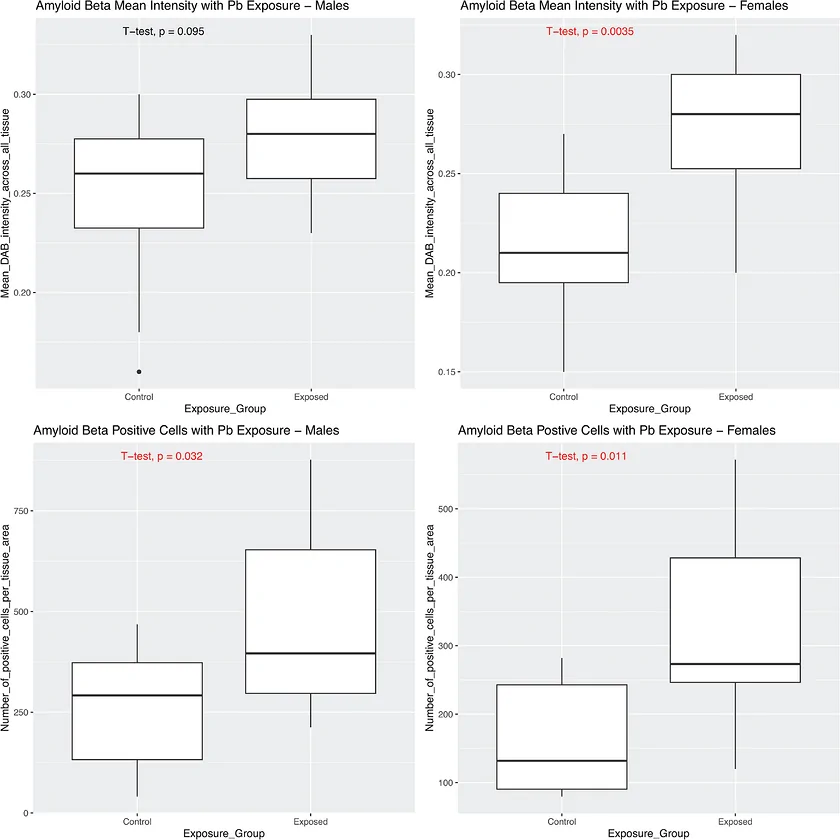
Quantification of diffuse amyloid beta (Aβ) staining. Mean intensity of diffuse Aβ immunohistochemical staining in males (A) and females (B), across the entire tissue area, and Aβ‐positive cells in males (C) and females (D). Statistical comparisons achieved using two‐sided *t* test (significance accepted at *P* < 0.05, highlighted in red). DAB, diaminobenzidine.

Aβ immunostaining was applied to Level 3 from each sample. This section contained the thalamus, hippocampus, and overlying cortex. Low to moderate intensity Aβ immunolabeling was present diffusely across the gray matter of the cerebrum and thalamus. White matter tracts (e.g., internal/external capsule) were mostly free from immunolabeling. Extracellular Aβ plaques, as in the positive control brain sample from a mouse model of AD, were not present (Table S3 in supporting information and Figure 2). Mean intensity of Aβ diffuse immunostaining across the entire tissue was higher in exposed samples relative to control for both males and females. In females, this difference reached statistical significance (*P* value = 0.0035) while in males it approached significance (*P* value = 0.095). More intense staining was noted in capillary walls throughout the brain, in serum (within capillaries), and in the mineralized thalamic loci (Figure 3, arrows). There was also moderate intensity cytoplasmic labeling within the cell bodies of large pyramidal neurons in the cerebral cortical layers, but not in the nuclei of these cells (Figure S2 in supporting information, arrows). Aβ immunostaining was also characterized according to the number of positive cells per tissue area. Pb‐exposed male tissues had, on average, 469 Aβ‐positive cells per tissue area compared to 258 in control male tissues (*P* value = 0.032), while Pb‐exposed female tissues had, on average, 320 Aβ‐positive cells per tissue area compared to 166 in control female tissues (*P* value = 0.011; Figure 4).

### 3.3 Perinatal Pb exposure is associated with metabolic changes in the brain and blood at PND21

The molecular results pertaining to Aβ prompted a question of whether Pb exposure perturbed molecular processes in a way that would support Aβ accumulation in the brain or other features related to AD. We assessed changes in the metabolome associated with perinatal Pb exposure during an early (PND21) and late‐life stage (18 months) in the cortex and plasma and found significant changes that were largely distinct by sex. At PND21, there were 124 biochemicals with differential (*P* value < 0.05) concentrations depending on Pb exposure status in the female cortex (55 increased with Pb exposure, 69 decreased) and 135 in the male cortex (54 increased with Pb exposure, 81 decreased). In PND21 plasma, 168 biochemicals displayed differential concentrations in female samples (105 increased with Pb exposure and 63 decreased) and 98 in male samples (24 increased with Pb exposure and 74 decreased; Table 1).

**TABLE 1:** Number of metabolites with differential concentrations with Pb exposure.

	Cortex			
	Female		Male	
	Increased	Decreased	Increased	Decreased
PND21	55	69	54	81
18 month	13	53	11	20

	Plasma			
	Female		Male	
	Increased	Decreased	Increased	Decreased
PND21	105	63	24	74
18 month	17	21	10	38

*Note*: Summary is stratified by tissue (cortex, plasma), time point (PND21, 18 months of age), and sex (male, female). Numbers of Pb‐associated metabolites are further characterized as increased (elevated in Pb‐exposed samples relative to control) or decreased (decreased in Pb‐exposed samples relative to control).

Abbreviations: Pb, lead; PND21, post‐natal day 21.

We observed elevated markers of oxidative stress in the PND21 cortex, including methionine sulfoxide (statistically significant in exposed female samples, FC = 1.22) and lanthionine (statistically significant in both sexes, *FC_female_* = 1.2 and *FC_male_* = 1.46). Glutathione, both the reduced (GSH) and oxidized (GSSG) forms, were lower in Pb exposed samples (GSH *FC_females_* = 0.56, *FC_males_* = 0.7; GSSG *FC_females_* = 0.87), suggesting an overall decrease in glutathione and a change in redox state.^27^ We observed alterations in biochemicals involved in one‐carbon metabolism, including serine (*FC_females_* = 1.14, *FC_males_* = 1.13). These observations are suggestive of increased oxidative stress^28^ in the Pb‐exposed group, with more pronounced changes in female mice. Plasma samples from PND21 also contained biochemicals with differential concentrations according to Pb exposure that are involved in the one‐carbon metabolism pathway, including glycine (FC = 1.14) and serine (FC = 1.24), though these differences were only significant in the bulk analysis and did not reach the statistical threshold in our sex‐stratified analyses. Betaine, a methyl group donor in transmethylation reactions,^29^ was notably reduced in Pb‐exposed female samples relative to control (FC = 0.83) and is of interest given that Pb exposure is known to disrupt methylation reactions.^30^

The PND21 time point also displayed differential concentrations of several biochemical markers of inflammation in both the cortex and plasma. In the cortex, we observed Pb exposure decreased several eicosanoids, which regulate various aspects of the inflammatory response.^31^ They included 5‐hydroxyeicosanoid (5‐HETE; *FC_female_* = 0.6, *FC_male_* = 0.65), 5‐keto‐eicosatetraenoic acid (5‐KETE; *FC_female_* = 0.58, *FC_male_* = 0.6), and 15‐KETE (*FC_male_* = 0.62). Interestingly, in the plasma, Pb exposure largely elevated eicosanoid metabolites including 12‐hydroxyeicosapentaenoic acid (12‐HEPE; *FC_female_* = 1.62, *FC_male_* = 2.03) and 12‐hydroxyeicosatetraenoic acid (12‐HETE; *FC_female_* = 2.05, *FC_male_* = 1.58), relative to control.

Sphingomyelin and ceramide metabolism was affected by Pb exposure at PND21, which is notable given their role in cell membranes and myelin in the nervous system.^32^ In the PND21 cortex, Pb exposure decreased in several ceramides and sphingomyelines relative to control, with FCs ranging from 0.69 to 0.94. This trend was not consistent in the corresponding plasma samples. While Pb exposure decreased several hexosylceramides relative to control (FC: 0.41–0.65) and these effects were largely seen in males, in plasma, Pb exposure elevated the vast majority of sphingomyelin relative to controls, with FCs in both female and male samples ranging from 1.25 to 1.65.

**FIGURE 5:**
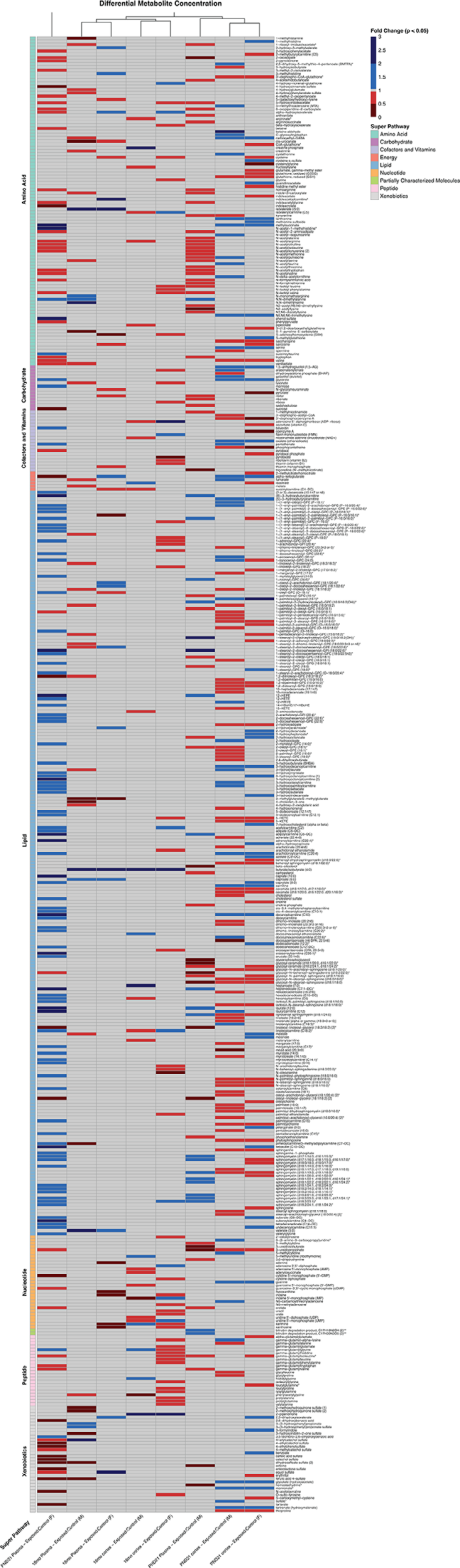
Summary of differential metabolite concentrations in murine cortex and plasma at post‐natal day 21 (PND21) and 18 months of age after perinatal lead (Pb) exposure. Differential metabolite levels between corresponding exposed and control tissues was calculated via two‐way analysis of variance, with significance accepted at *P* < 0.05 (pictured results include *P* < 0.005). Metabolites are annotated according to pathway (amino acid, carbohydrate, cofactors and vitamins, energy, lipid, nucleotide, partially characterized molecules, peptide, xenobiotics) and fold change in metabolite in level is indicated by cell color (red = decreased level in exposed vs. control tissue, blue = increased level in exposed vs. control tissue, gray = no difference).

Pb exposure was also associated with changes in fatty acid metabolism in the PND21 cortex and plasma, with female samples driving this trend in both tissues. In the cortex, FCs in medium chain fatty acids as well as dicarboxylate and monohydroxy fatty acid metabolites ranged from 1.28 to 1.6. Pb exposure decreased only one fatty acid metabolite relative to control (2‐hydroxyadipate; *FC_female_* = 0.52, *FC_male_* = 0.54). This trend was more pronounced in the plasma samples, with FCs of medium and long chain fatty acids as well as long chain polyunsaturated fatty acids and dicarboxylate fatty acid metabolites ranging from 1.3 to 2.69. A summary of metabolite FCs and significance at PND21 can be found in Figure 5 and full results shown in Table S4 in supporting information (cortex) and Table S5 in supporting information (plasma).

### 3.4 Pb‐associated metabolites at PND21 associated with lipid metabolism and neurological conditions

**FIGURE 6:**
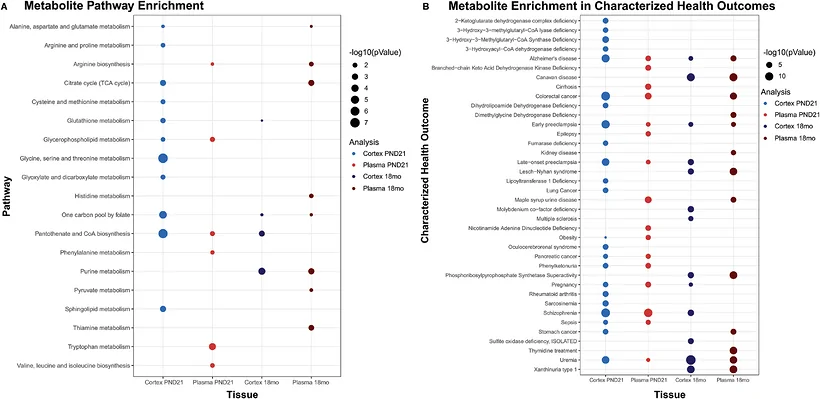
Metabolite pathway and characterized health outcome enrichment analysis. Lists of metabolites (compound names) with significant (*P* < 0.05) differential concentration in control versus exposed tissues (cortex, plasma) for each time point (post‐natal day 21 [PND21], 18 months) were evaluated and input into the Pathway Analysis tool on MetaboAnalyst. Name/ID standardization and parameter selection (Hypergeometric Test, reference metabolome: Mus musculus) were performed using the MetaboAnalyst online platform and significant pathway enrichment was accepted at *P* < 0.05. Results are visualized according to tissue (PND21 cortex, PND21 plasma, 18 months cortex, and 18 months plasma) with point size corresponding to significance (A). Lists of metabolites (compound names) with significant (*P* < 0.05) differential levels in control versus exposed tissues (cortex, plasma) for each time point (PND21, 18 months) were input into the Enrichment Analysis tool on MetaboAnalyst. Name/ID standardization and parameter selection (Disease Signature: Blood) were performed using the MetaboAnalyst online platform and significant pathway enrichment was accepted at *P* < 0.05. Results are visualized according to tissue (PND21 cortex, PND21 plasma, 18 months cortex, and 18 months plasma) with point size corresponding to significance (B).

We used MetaboAnalyst to perform pathway and health outcome enrichment analysis of the metabolites significantly associated with Pb exposure at PND21. There was significant enrichment for amino acid biosynthesis and metabolism in the cortex (12 amino acid pathways enriched) and, to a lesser degree, in the plasma (6 amino acid pathways enriched). Lipid metabolism was also enriched in our results at PND21 (glycerophospholipid metabolism in both cortex and plasma, sphingolipid metabolism in cortex alone) as well as energy metabolism, with pathways including the citrate cycle and CoA biosynthesis enriched in the cortex and glyoxylate/dicarboxylate metabolism enriched in both cortex and plasma. A full summary of enriched metabolic pathways is available in Figure 6A.

We next assessed Pb‐associated metabolite enrichment with characterized health outcomes using MetaboAnalyst's repository built from 15 libraries consisting of > 13,000 biologically meaningful metabolite sets from primarily human studies and encompassing > 1500 chemical classes.^33^ Pb‐associated metabolites were enriched for neurodegenerative outcomes, including AD (cortex and plasma), dementia (cortex), as well as numerous other neurological conditions including autism (cortex), epilepsy (plasma), peripheral neuropathy (cortex), schizophrenia (cortex and plasma), and stroke (cortex). Additional general categories of health outcomes that were apparent at the PND21 time point across both tissues included cancer (e.g., leukemia, colorectal, lung, pancreatic, stomach) and hypertensive conditions including early and late preeclampsia, in addition to pregnancy more broadly. A full summary of enriched health outcomes is provided in Figure 6B.

### 3.5 Perinatal Pb exposure associated with limited metabolic changes at 18 months

In aged mice, Pb exposure was associated with differences in fewer biochemicals in either the cortex or plasma compared to mice at PND21. At 18 months, Pb exposure was associated with 66 biochemicals in the female cortex (13 increased with Pb exposure, 53 decreased) and 31 in the male cortex (11 increased with Pb exposure, 20 decreased). In 18‐month plasma, 38 biochemicals displayed differential concentrations with Pb exposure in female samples (17 increased with Pb exposure and 21 decreased) and 48 in male samples (10 increased with Pb exposure and 38 decreased; Table 1). This difference in the total number of metabolomic changes between time points may be partially explained by increased variability in metabolism as animals age and greater dysregulation of metabolic processes in general that may make it more difficult to detect changes associated with just the exposure in this study.^34^

There was little overlap in Pb‐associated metabolites between time points. At 18 months, we saw some markers of oxidative stress in the cortex, with significant results limited to Pb‐exposed female samples relative to control, including glycine (FC = 0.87) and 4‐hydroxy‐nonenal‐glutathione (FC = 1.73). We observed no changes in glutathione metabolism in the plasma, except opthalamate, which was significant only in the combined analysis across both sexes (FC = 0.56). We also observed limited changes in inflammation markers such as eicosanoids in both the cortex and the plasma. In cortex, 5‐HETE was reduced with Pb exposure in females (FC = 0.59), while in plasma 15‐HETE was reduced with Pb exposure in the combined analysis of female and male samples (FC = 0.35).

Sphingomyelin metabolites were also largely unchanged with Pb exposure at 18 months. In the cortex, one such metabolite, sphingomyelin (d18:2/23:1), was enriched in Pb‐exposed female cortex (FC = 1.91) and one ceramide metabolite, N‐βehenoyl‐sphingadienine (d18:2/22:0), was decreased with Pb exposure in female samples (FC = 0.51). In the plasma there were no significant changes in any sphingomyelin or ceramide metabolites in either sex or in the combined analysis. We did observe other changes in lipid metabolism more broadly in the cortex at 18 months, with most changes limited to female samples and largely decreased metabolite concentrations with Pb exposure relative to control. In the cortex, these included several endocannabinoids, with FCs ranging from 0.49 to 0.74 and lysophospholipids, with FCs ranging from 0.74 to 0.82. In the plasma, we observed negative associations between Pb and several dicarboxylate fatty acid metabolites (FC: 0.24–0.83) as well as phosphatidylethanolamine metabolites. Pb associations in the latter class were predominantly positive in female samples (FC: 1.32–1.53) and negative in males (FC: 0.52–0.72). The limited instances of consistent positive associations between Pb exposure and lipid metabolites in both female and male samples were much greater in magnitude, including butyrate/isobutyrate (4:0; cortex: *FC_female_* = 2.27, *FC_male_* = 5.14, plasma: *FC_female_* = 2.7, *FC_male_* = 2.67) as well as valerate (*FC_female_* = 1.95, *FC_male_* = 2.29) and caproate (*FC_female_* = 1.21, *FC_male_* = 1.35) in plasma. A summary of metabolite FCs and significance at 18 months can be found in Figure 5 and Table S6 in supporting information (cortex) and Table S7 in supporting information (plasma).

### 3.6 Pb‐associated metabolites at 18 months were enriched for neurodegenerative and metabolic conditions

The relatively limited number of metabolites significantly associated with Pb exposure at the 18‐month time point correlated with fewer enriched pathways in the cortex (*n* = 4) compared to the PND21 findings (*n* = 11). In the cortex, there was some overlap with the PND21 time point, including enrichment for CoA biosynthesis, one carbon pool by folate and glutathione metabolism. A pathway unique to the aged time point, significant in both cortex and plasma, was purine metabolism. In the 18‐month plasma, we observed enrichment for the metabolism of six amino acids as well as metabolites involved in the citrate cycle (Figure 6A and Table S8 in supporting information).

When assessing for metabolite enrichment in characterized health outcomes, we again observed significant overlap with neurodegenerative conditions including AD and dementia in both the cortex and plasma, as well as multiple sclerosis (cortex). We observed more limited enrichment for hypertensive conditions (early and late preeclampsia and hyperoxalemia) as well as cancers. Pb‐associated metabolites were more likely to be enriched for metabolic conditions at 18 months and limited to the cortex, including lactic acidosis, Fanconi syndrome, hyperglycemia, and peroxisomal disorders (Figure 6B and Table S9 in supporting information).

### 3.7 Identification of candidate metabolite biomarkers of Pb exposure in the cortex and plasma

**FIGURE 7:**
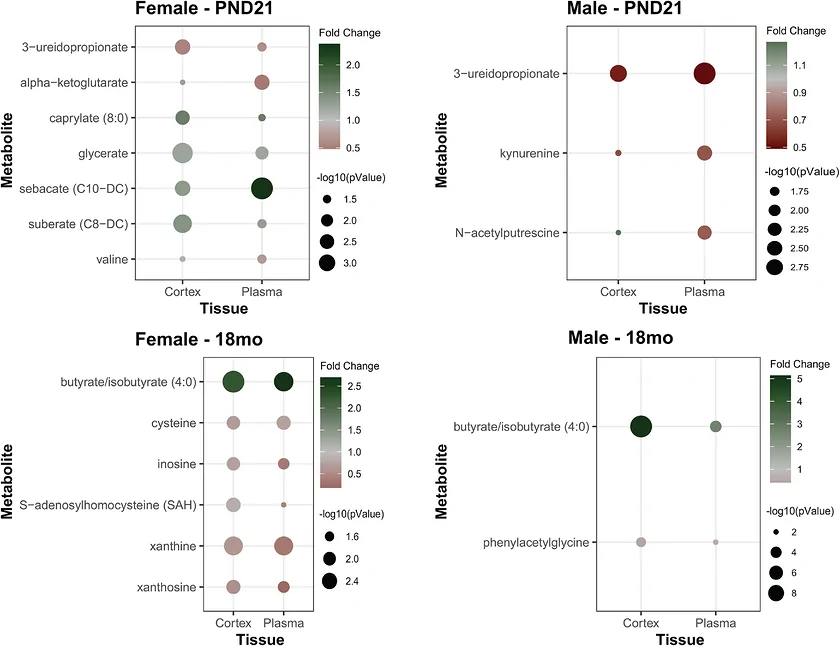
Metabolites affected by perinatal lead (Pb) exposure in both cortex and plasma. Significant (*P* < 0.05) differences in metabolite concentration between exposed and control tissues were calculated using two‐way analysis of variance, stratified by sex (male and female) at post‐natal day 21 (PND1) and 18 months (18 mo) of age. Results are visualized according to the direction of effect (green = positive fold change or red = negative fold change in Pb‐exposed mice relative to control), with point size corresponding to significance.

An additional objective of this work was to identify Pb‐associated biochemicals that were present in both cortex and plasma for a given time point and sex combination to assess the utility of plasma metabolites to serve as biomarkers of the effect of Pb in the brain. We have previously explored the epigenome and found limited but biologically relevant signatures of Pb exposure in both the cortex and blood.^35^ Here, we looked for biochemicals with the same direction of significant association with Pb in both cortex and plasma samples, stratified by sex and age. At both time points, females had more consistency across tissues than males. At PND21, female cortex and plasma had seven Pb‐associated metabolites in common (3‐uriedopropionate, alpha‐ketoglutarate, caprylate, glycerate, sebacate, suberate, and valine) while males had three (3‐ureidpropoionate, kynurenine, and N‐acetylputrescine). Both sexes had decreased concentrations of 3‐uriedoproionate in cortex and plasma, but no other consistency between the sexes was identified. At 18 months, female cortex and plasma had six Pb‐associated biochemicals in common (butyrate, cysteine, inosine, S‐adenosylhomocysteine, xanthine, and xanthosine) while males again only had two (butyrate and phenylacetylglycine). The two sexes both displayed elevated concentrations of butyrate/isobutryate with Pb exposure. While the total number of candidate metabolite biomarkers was the same in each sex across the two time points, there were no Pb‐associated biochemicals present in the same sex and across both tissues at both PND21 and 18 months (Figure 7).

## 4 DISCUSSION

We assessed AD‐relevant molecular and metabolic changes in mice after perinatal Pb exposure and found alterations in later‐in‐life levels of diffuse Aβ and Aβ‐positive cells throughout the brain, with higher staining intensity near vasculature and stronger trends in female mice. In humans with AD, Aβ accumulates and forms neurotoxic plaques.^36^ C57BL/6J mice are resistant to many hallmarks of AD, including the development of Aβ plaques,^37^ which likely explains why we did not observe plaques.

Aβ is produced via enzymatic cleavage of amyloid precursor protein (APP).^38^ APP has dual roles in the brain. In the non‐amyloidogenic pathway, APP is cleaved by α‐secretase, producing neuroprotective fragments such as sAPPα, which promotes growth^39^ and protects against excitotoxicity.^40^ Amyloidogenic cleavage of APP by β‐secretase and γ‐secretase produces soluble C‐terminal β fragments, which are further processed into Aβ.^41^ Given the role of non‐amyloidogenic processing of APP and sAPPα in responding to neuronal death^42^ and oxidative stress response,^43^ two outcomes caused by Pb,^44^, ^45^ elevated Aβ could be a byproduct of the brain's attempt to mitigate Pb neurotoxicity. Theoretically, additional APP production and processing may be triggered by Pb's neurotoxic effects and while processing by α‐secretase into sAPPα is the main response pathway, additional cleavage by background levels of β‐secretase and γ‐secretase drove up diffuse levels of Aβ. Validating this theory would require complementary analyses of APP specifically, along with secretase enzymes and sAPPα. It would be informative to assess whether differences in diffuse Aβ levels after Pb exposure were due to changes in deposition, clearance, and/or vascular accumulation.

To follow up on histopathological findings, we investigated metabolic changes in the brain and plasma at the cessation of exposure (PND21) and 18 months of age to determine: (1) whether Pb modifies metabolism to promote diffuse Aβ increases, (2) whether exposure‐associated metabolic changes are enriched for AD pathways, and (3) whether Pb‐associated metabolic changes in the brain are mirrored in the plasma to identify biomarkers of exposure and/or effects.

We observed Pb‐associated alterations in many metabolites implicated in oxidative stress and inflammation. These two outcomes are documented after Pb exposure, as Pb increases reactive oxygen species^46^ and promotes cytokine activation^47^ and prostaglandins.^48^ Oxidative stress and inflammation are both hallmarks of neurodegenerative disease.^49^ Pb‐associated metabolites in these pathways were present to a greater degree at PND21 compared to 18 months of age. These differences between time points may be partially explained by increased variability in metabolism as animals age.^34^ Oxidative stress during neurodevelopment is associated with elevated AD risk through aberrant gene expression regulation,^50^ damage to cellular membranes,^51^ and increased neural cell death.^52^ Neurodevelopmental inflammation is suspected to prime microglia and promote chronic, low‐grade inflammation,^15^ which has a reinforcing relationship with Aβ plaque formation.^53^ Our results support the hypothesis that neurodevelopmental perturbations compromise neural systems through metabolic alterations including oxidative stress and inflammation, which may increase AD risk. To test this theory more directly, future work should quantify oxidative stress and neuroinflammation alongside features of AD, to assess whether Pb‐associated outcomes result from these molecular perturbations.

The most notable finding from the metabolomics was the overwhelming number of lipid metabolites impacted by Pb. Lipids play important roles in the brain, as structural components of cell and vesicle membranes^54^ and myelin,^55^ and acting as energy sources.^56^ Butyrate/isobutyrate had a strong relationship with Pb at 18 months in both sexes and tissues. These short‐chain fatty acids are produced through dietary fiber fermentation^57^ and reduce neuroinflammation^58^ and promote neural growth.^59^ It is plausible that elevated butyrate is a compensatory response to Pb‐induced oxidative stress and inflammation. Butyrate has been explored as a therapeutic agent for neurodegenerative disease.^60^ Assessments of butyrate's ability to ameliorate Pb‐associated oxidative stress and inflammation, and features such as Aβ plaques, in human AD‐relevant experimental models would expand our understanding of the utility of this metabolite in AD prevention and therapeutics.

Sphingomyelins and ceramides were another class of lipid metabolites commonly altered by Pb exposure, particularly at PND21, when they were largely decreased in the cortex and increased in the plasma. These compounds are key components of cell membranes in the nervous system, where they maintain membrane structure and function in cell signaling. During neurodevelopment, sphingomyelins are important for neuronal growth, development, and maintenance, including regulating differentiation, neurite outgrowth, and synaptogenesis.^61^ The decrease of these metabolites in the cortex may be caused by Pb's induction of oxidative stress, which could degrade sphingomyelins and ceramides. The increase of these metabolites in the plasma may reflect disrupted blood–brain barrier function by early life Pb exposure,^62^ with increased permeability causing leakage of sphingomyelins into circulation. Elevated plasma ceramides and sphingomyelin have been associated with increased AD risk, with variation depending on sex, over 10 to 15 years of follow‐up.^63^ Thus, plasma ceramides and sphingomyelin may represent a circulating biomarker which reflects toxic effects of developmental Pb exposure in the brain as well as AD risk later in life, and deserve additional investigation.

Pb‐associated metabolites were also enriched in amino acid metabolism. Amino acid catabolism is a proposed feature of AD, resulting from reduced glycolysis and reliance on an alternative energy source.^64^ A deeper analysis of Pb exposure and changes in amino acid concentrations and their catabolism would shed light on whether Pb contributes to AD through this pathway. Interestingly, purine metabolism was also indicated in our pathway analysis. Purine metabolism is activated in the DNA damage repair response,^65^ and DNA damage is a feature of both Pb exposure^66^ and AD.^67^ Continued evaluation of this relationship would benefit from models that can capture AD molecular features to assess associations between molecular pathways and pathological outcomes, as well as from methods for determining the location of elevated brain DNA damage^68^ to determine whether Pb‐associated damage occurs preferentially in brain regions most affected by AD.

While we observed Pb‐associated changes in several classes of metabolites previously shown to be related to AD outcomes and pathology, we did not explicitly test any of these relationships. As such, it would be prudent for future work to interrogate direct links between metabolite changes and AD‐molecular features such as Aβ, which would support our understanding of how these metabolic pathways mechanistically contribute to AD development and progression, and how toxicants drive AD risk through metabolic changes. Additionally, an assessment of behavioral and cognitive outcomes would add to our understanding of how neurotoxicants drive neurodegenerative disease. Future work in C57BL/6J mice, as well as AD‐specific models such as 5xFAD mice, could fill this gap.

The only two health outcomes that were enriched for Pb‐associated metabolites across both tissues and time points were AD and early preeclampsia. This reinforces our hypothesis that Pb exposure is a modifiable risk factor for AD and, despite our inability to examine Aβ plaques in the C57BL/6J model, the metabolome responds to this toxicant in ways that mirror the health outcome of interest. Preeclampsia is also of interest for future work given its relationship to neurodegenerative outcomes, as they are suspected to have mechanisms in common, including increased inflammation and levels of Aβ.^69^ There is a tentative association between preeclampsia and cognitive impairment over 35 to 45 years of follow‐up.^70^ Pb is also associated with preeclampsia^71^ and proposed mechanisms for this association mirror that of AD, including oxidative stress^72^ and compromised vasculature.^73^ Our results are exploratory in nature, and should not be taken to mean Pb is indeed relevant for preeclampsia risk, given that this health outcome was not tested. However, given the known association between preeclampsia and cognitive deficits, concurrent in vivo evaluations of both AD and preeclampsia after Pb exposure would test toxicant mechanisms common to both outcomes and help infer whether Pb‐driven preeclampsia exacerbates AD risk.

We observed a limited set of Pb‐associated metabolites in both cortex and blood plasma, with 3‐ureidopropionate common in both males and females at PND21 and butyrate/isobutyrate common across sexes at 18 months. These limited and specific Pb‐associated metabolites detected in both tissues suggest that neurotoxicology metabolomics should consider sex and age, as these impact metabolism. Indeed, metabolic differences have been well documented between the sexes and with age due to hormone signaling,^74^, ^75^ anatomical changes,^76^ and tissue‐level shifts such as adipose composition.^77^

Developmental exposure to human‐relevant levels of Pb produce persistent, late‐life AD‐related molecular and metabolic alterations in the cortex, detectable > 17 months after exposure ceased. These findings provide experimental evidence that an early‐life environmental toxicant can durably reprogram trajectories of brain aging and vulnerability to neurodegenerative disease, supporting a developmental origins framework for AD that is difficult to infer from predominantly cross‐sectional human studies. The convergence of oxidative stress, neuroinflammation, and disrupted lipid and amino acid metabolism suggest plausible, interacting mechanisms through which Pb may lower resilience to age‐associated proteostasis and synaptic dysfunction, and highlights pathway‐level signatures that could be leveraged as biomarkers of risk before clinical manifestation. This work underscores the importance of considering timing of exposure across the life course, identifies mechanistically grounded targets for intervention, and strengthens the rationale for primary prevention by continuing to reduce Pb exposure, particularly during pregnancy and early development, to mitigate later life dementia burden.

## CONFLICT OF INTEREST STATEMENT

The authors declare no conflicts of interest. Author disclosures are available in the Supporting Information.

## CONSENT STATEMENT

No human subjects were used in this project and thus, consent was not necessary.

## Supporting information



Supporting Information

Supporting Information

Supporting Information

Supporting Information

Supporting Information

Supporting Information

Supporting Information

Supporting Information

Supporting Information

Supporting Information

Supporting Information

Supporting Information

Supporting Information

## References

[1] 2024 Alzheimer's disease facts and figures. Alzheimers Dement . 2024;20(5):3708‐3821. doi:10.1002/alz.13809 10.1002/alz.13809 PMC11095490 38689398

[2] Rajan KB , Weuve J , Barnes LL , McAninch EA , Wilson RS , Evans DA . Population estimate of people with clinical Alzheimer's disease and mild cognitive impairment in the United States (2020‐2060). Alzheimers Dement. 2021;17(12):1966‐1975. doi:10.1002/alz.12362 34043283 10.1002/alz.12362 PMC9013315

[3] Hebert LE , Weuve J , Scherr PA , Evans DA . Alzheimer disease in the United States (2010‐2050) estimated using the 2010 census. Neurology. 2013;80(19):1778‐1783. doi:10.1212/WNL.0b013e31828726f5 23390181 10.1212/WNL.0b013e31828726f5 PMC3719424

[4] Bellenguez C , Küçükali F , Jansen IE , et al. New insights into the genetic etiology of Alzheimer's disease and related dementias. Nat Genet. 2022;54(4):412‐436. doi:10.1038/s41588‐022‐01024‐z 35379992 10.1038/s41588-022-01024-z PMC9005347

[5] Bakulski KM , Seo YA , Hickman RC , et al. Heavy metals exposure and Alzheimer's disease and related dementias. J Alzheimers Dis. 2020;76(4):1215‐1242. doi:10.3233/JAD‐200282 32651318 10.3233/JAD-200282 PMC7454042

[6] Swerdlow RH . Is aging part of Alzheimer's disease, or is Alzheimer's disease part of aging?. Neurobiol Aging. 2007;28(10):1465‐1480. doi:10.1016/j.neurobiolaging.2006.06.021 16876913 10.1016/j.neurobiolaging.2006.06.021

[7] Fleming TP , Sun C , Denisenko O , et al. Environmental exposures around conception: developmental pathways leading to lifetime disease risk. Int J Environ Res Public Health. 2021;18(17):9380. doi:10.3390/ijerph18179380 34501969 10.3390/ijerph18179380 PMC8431664

[8] Borghi F , Mazzucchelli LA , Campagnolo D , et al. Retrospective exposure assessment methods used in occupational human health risk assessment: a systematic review. Int J Environ Res Public Health. 2020;17(17):6190. doi:10.3390/ijerph17176190 32858967 10.3390/ijerph17176190 PMC7504303

[9] Radulescu CI , Cerar V , Haslehurst P , Kopanitsa M , Barnes SJ . The aging mouse brain: cognition, connectivity and calcium. Cell Calcium. 2021;94:102358. doi:10.1016/j.ceca.2021.102358 33517250 10.1016/j.ceca.2021.102358

[10] Ruckart PZ , Jones RL , Courtney JG , et al. Update of the blood lead reference value—United States, 2021. MMWR Morb Mortal Wkly Rep. 2021;70(43):1509‐1512. doi:10.15585/mmwr.mm7043a4 34710078 10.15585/mmwr.mm7043a4 PMC8553025

[11] McFarland MJ , Hauer ME , Reuben A . Half of US population exposed to adverse lead levels in early childhood. Proc Natl Acad Sci U S A. 2022;119(11):e2118631119. doi:10.1073/pnas.2118631119 35254913 10.1073/pnas.2118631119 PMC8931364

[12] Wang X , Bakulski KM , Walker E , et al. Exposure to lead and incidence of Alzheimer's disease and all‐cause dementia in the United States. Alzheimers Dement. 2026;22(2):e71075. doi:10.1002/alz.71075 41676992 10.1002/alz.71075 PMC12895363

[13] Essers E , Binter AC , Neumann A , White T , Alemany S , Guxens M . Air pollution exposure during pregnancy and childhood, APOE ε4 status and Alzheimer polygenic risk score, and brain structural morphology in preadolescents. Environ Res. 2023;216(Pt 2):114595. doi:10.1016/j.envres.2022.114595 36257450 10.1016/j.envres.2022.114595

[14] Kisby GE , Spencer PS . Genotoxic damage during brain development presages prototypical neurodegenerative disease. Front Neurosci. 2021;15:752153. doi:10.3389/fnins.2021.752153 34924930 10.3389/fnins.2021.752153 PMC8675606

[15] Kopalli SR , Vadia N , Varma P , et al. Neurodevelopmental origins of neurodegeneration: a lifespan perspective on brain vulnerability. Brain Res. 2026;1873:150134. doi:10.1016/j.brainres.2025.150134 41456636 10.1016/j.brainres.2025.150134

[16] Ambeskovic M , Hopkins G , Hoover T , Joseph JT , Montina T , Metz GAS . Metabolomic signatures of Alzheimer's disease indicate brain region‐specific neurodegenerative progression. Int J Mol Sci. 2023;24(19):14769. doi:10.3390/ijms241914769 37834217 10.3390/ijms241914769 PMC10573054

[17] Batra R , Arnold M , Wörheide MA , et al. The landscape of metabolic brain alterations in Alzheimer's disease. Alzheimers Dement. 2023;19(3):980‐998. doi:10.1002/alz.12714 35829654 10.1002/alz.12714 PMC9837312

[18] Faulk C , Barks A , Liu K , Goodrich JM , Dolinoy DC . Early‐life lead exposure results in dose‐ and sex‐specific effects on weight and epigenetic gene regulation in weanling mice. Epigenomics. 2013;5(5):487‐500. doi:10.2217/epi.13.49 24059796 10.2217/epi.13.49 PMC3873735

[19] Committee on Obstetric Practice. Committee opinion No. 533: lead screening during pregnancy and lactation. Obstet Gynecol. 2012;120(2 Pt 1):416‐420. doi:10.1097/AOG.0b013e31826804e8 22825110 10.1097/AOG.0b013e31826804e8

[20] Svoboda LK , Neier K , Wang K , et al. Tissue and sex‐specific programming of DNA methylation by perinatal lead exposure: implications for environmental epigenetics studies. Epigenetics. 2021;16(10):1102‐1122. doi:10.1080/15592294.2020.1841872 33164632 10.1080/15592294.2020.1841872 PMC8510611

[21] Bolon B , Garman RH , Pardo ID , et al. STP position paper: recommended practices for sampling and processing the nervous system (brain, spinal cord, nerve, and eye) during nonclinical general toxicity studies. Toxicol Pathol. 2013;41(7):1028‐1048. doi:10.1177/0192623312474865 23475559 10.1177/0192623312474865

[22] Bankhead P , Loughrey MB , Fernández JA , et al. QuPath: open source software for digital pathology image analysis. Sci Rep. 2017;7(1):16878. doi:10.1038/s41598‐017‐17204‐5 29203879 10.1038/s41598-017-17204-5 PMC5715110

[23] Pang Z , Lu Y , Zhou G , et al. MetaboAnalyst 6.0: towards a unified platform for metabolomics data processing, analysis and interpretation. Nucleic Acids Res. 2024;52(W1):W398‐W406. doi:10.1093/nar/gkae253 38587201 10.1093/nar/gkae253 PMC11223798

[24] Bradley AE , Bolon B , Butt MT , et al. Proliferative and nonproliferative lesions of the rat and mouse central and peripheral nervous systems: new and revised INHAND terms. Toxicol Pathol. 2020;48(7):827‐844. doi:10.1177/0192623320951154 32912053 10.1177/0192623320951154

[25] Kaufmann W , Bolon B , Bradley A , et al. Proliferative and nonproliferative lesions of the rat and mouse central and peripheral nervous systems. Toxicol Pathol. 2012;40(4):87S‐157S. doi:10.1177/0192623312439125 22637737 10.1177/0192623312439125

[26] Garman RH . Histology of the central nervous system. Toxicol Pathol. 2011;39(1):22‐35. doi:10.1177/0192623310389621 21119051 10.1177/0192623310389621

[27] Rebrin I , Sohal RS . Pro‐oxidant shift in glutathione redox state during aging. Adv Drug Deliv Rev. 2008;60(13‐14):1545‐1552. doi:10.1016/j.addr.2008.06.001 18652861 10.1016/j.addr.2008.06.001 PMC2585506

[28] Wang F , Zhou H , Deng L , Wang L , Chen J , Zhou X . Serine deficiency exacerbates inflammation and oxidative stress via microbiota‐gut‐brain axis in d‐galactose‐induced aging mice. Mediators Inflamm. 2020;2020:5821428. doi:10.1155/2020/5821428 32189994 10.1155/2020/5821428 PMC7071807

[29] Craig SAS . Betaine in human nutrition. Am J Clin Nutr. 2004;80(3):539‐549. doi:10.1093/ajcn/80.3.539 15321791 10.1093/ajcn/80.3.539

[30] Senut MC , Sen A , Cingolani P , Shaik A , Land SJ , Ruden DM . Lead exposure disrupts global DNA methylation in human embryonic stem cells and alters their neuronal differentiation. Toxicol Sci. 2014;139(1):142‐161. doi:10.1093/toxsci/kfu028 24519525 10.1093/toxsci/kfu028 PMC4023291

[31] Ebright B , Assante I , Poblete RA , et al. Eicosanoid lipidome activation in post‐mortem brain tissues of individuals with APOE4 and Alzheimer's dementia. Alzheimers Res Ther. 2022;14(1):152. doi:10.1186/s13195‐022‐01084‐7 36217192 10.1186/s13195-022-01084-7 PMC9552454

[32] McCluskey G , Donaghy C , Morrison KE , McConville J , Duddy W , Duguez S . The role of sphingomyelin and ceramide in motor neuron diseases. J Pers Med. 2022;12(9):1418. doi:10.3390/jpm12091418 36143200 10.3390/jpm12091418 PMC9501626

[33] Xia J , Sinelnikov IV , Han B , Wishart DS . MetaboAnalyst 3.0–making metabolomics more meaningful. Nucleic Acids Res. 2015;43(W1):W251‐257. doi:10.1093/nar/gkv380 25897128 10.1093/nar/gkv380 PMC4489235

[34] Qian S , Ba Y , Xue L , et al. Age‐related changes of human brain metabolism. Eur J Nucl Med Mol Imaging. 2025;52(9):3412‐3423. doi:10.1007/s00259‐025‐07211‐4 40137976 10.1007/s00259-025-07211-4

[35] Morgan RK , Wang K , Svoboda LK , et al. Effects of developmental lead and phthalate exposures on DNA methylation in adult mouse blood, brain, and liver: a focus on genomic imprinting by tissue and sex. Environ Health Perspect. 2024;132(6):67003. doi:10.1289/EHP14074 38833407 10.1289/EHP14074 PMC11166413

[36] Mucke L , Selkoe DJ . Neurotoxicity of amyloid β‐protein: synaptic and network dysfunction. Cold Spring Harb Perspect Med. 2012;2(7):a006338. doi:10.1101/cshperspect.a006338 22762015 10.1101/cshperspect.a006338 PMC3385944

[37] Meyer‐Luehmann M , Coomaraswamy J , Bolmont T , et al. Exogenous induction of cerebral beta‐amyloidogenesis is governed by agent and host. Science. 2006;313(5794):1781‐1784. doi:10.1126/science.1131864 16990547 10.1126/science.1131864

[38] Zhang C , Browne A , Divito JR , et al. Amyloid‐β production via cleavage of amyloid‐β protein precursor is modulated by cell density. J Alzheimers Dis. 2010;22(2):683‐984. doi:10.3233/JAD‐2010‐100816 20847415 10.3233/JAD-2010-100816 PMC3272776

[39] Gakhar‐Koppole N , Hundeshagen P , Mandl C , et al. Activity requires soluble amyloid precursor protein alpha to promote neurite outgrowth in neural stem cell‐derived neurons via activation of the MAPK pathway. Eur J Neurosci. 2008;28(5):871‐882. doi:10.1111/j.1460‐9568.2008.06398.x 18717733 10.1111/j.1460-9568.2008.06398.x

[40] Mattson MP , Cheng B , Culwell AR , Esch FS , Lieberburg I , Rydel RE . Evidence for excitoprotective and intraneuronal calcium‐regulating roles for secreted forms of the beta‐amyloid precursor protein. Neuron. 1993;10(2):243‐254. doi:10.1016/0896‐6273(93)90315‐i 8094963 10.1016/0896-6273(93)90315-i

[41] Zhang X , Song W . The role of APP and BACE1 trafficking in APP processing and amyloid‐β generation. Alzheimers Res Ther. 2013;5(5):46. doi:10.1186/alzrt211 24103387 10.1186/alzrt211 PMC3978418

[42] Araki W , Kitaguchi N , Tokushima Y , et al. Trophic effect of beta‐amyloid precursor protein on cerebral cortical neurons in culture. Biochem Biophys Res Commun. 1991;181(1):265‐271. doi:10.1016/s0006‐291x(05)81412‐3 1958195 10.1016/s0006-291x(05)81412-3

[43] Goodman Y , Mattson MP . Secreted forms of beta‐amyloid precursor protein protect hippocampal neurons against amyloid beta‐peptide‐induced oxidative injury. Exp Neurol. 1994;128(1):1‐12. doi:10.1006/exnr.1994.1107 8070512 10.1006/exnr.1994.1107

[44] Bandaru LJM , Murumulla L , BL C , KP D , Challa S . Exposure of combination of environmental pollutant, lead (Pb) and β‐amyloid peptides causes mitochondrial dysfunction and oxidative stress in human neuronal cells. J Bioenerg Biomembr. 2023;55(1):79‐89. doi:10.1007/s10863‐023‐09956‐9 36637735 10.1007/s10863-023-09956-9

[45] Niu Y , Pan Y , Wang Y , et al. Ferroptosis induced by Pb exposure causes parkinson's disease‐related dopaminergic neuronal death. Environ Sci Technol. 2025;59(47):25186‐25199. doi:10.1021/acs.est.5c11674 41160771 10.1021/acs.est.5c11674

[46] Lin HW , Lee HL , Shen TJ , et al. Pb(NO3)2 induces cell apoptosis through triggering of reactive oxygen species accumulation and disruption of mitochondrial function via SIRT3/SOD2 pathways. Environ Toxicol. 2024;39(3):1294‐1302. doi:10.1002/tox.24019 37948429 10.1002/tox.24019

[47] Chibowska K , Baranowska‐Bosiacka I , Falkowska A , Gutowska I , Goschorska M , Chlubek D . Effect of Lead (Pb) on Inflammatory Processes in the Brain. Int J Mol Sci. 2016;17(12):2140. doi:10.3390/ijms17122140 27999370 10.3390/ijms17122140 PMC5187940

[48] Chetty CS , Vemuri MC , Reddy GR , Suresh C . Protective effect of 17‐beta‐estradiol in human neurocellular models of lead exposure. Neurotoxicology. 2007;28(2):396‐401. doi:10.1016/j.neuro.2006.03.012 16678263 10.1016/j.neuro.2006.03.012

[49] Wilson DM , Cookson MR , Van Den Bosch L , Zetterberg H , Holtzman DM , Dewachter I . Hallmarks of neurodegenerative diseases. Cell. 2023;186(4):693‐714. doi:10.1016/j.cell.2022.12.032 36803602 10.1016/j.cell.2022.12.032

[50] Weitzman SA , Turk PW , Milkowski DH , Kozlowski K . Free radical adducts induce alterations in DNA cytosine methylation. Proc Natl Acad Sci U S A. 1994;91(4):1261‐1264. doi:10.1073/pnas.91.4.1261 8108398 10.1073/pnas.91.4.1261 PMC43137

[51] Itri R , Junqueira HC , Mertins O , Baptista MS . Membrane changes under oxidative stress: the impact of oxidized lipids. Biophys Rev. 2014;6(1):47‐61. doi:10.1007/s12551‐013‐0128‐9 28509959 10.1007/s12551-013-0128-9 PMC5425709

[52] Xie H , Hou S , Jiang J , Sekutowicz M , Kelly J , Bacskai BJ . Rapid cell death is preceded by amyloid plaque‐mediated oxidative stress. Proc Natl Acad Sci U S A. 2013;110(19):7904‐7909. doi:10.1073/pnas.1217938110 23610434 10.1073/pnas.1217938110 PMC3651444

[53] Alasmari F , Alshammari MA , Alasmari AF , Alanazi WA , Alhazzani K . Neuroinflammatory cytokines induce amyloid beta neurotoxicity through modulating amyloid precursor protein levels/metabolism. Biomed Res Int. 2018;2018:3087475. doi:10.1155/2018/3087475 30498753 10.1155/2018/3087475 PMC6222241

[54] Su H , Rustam YH , Masters CL , et al. Characterization of brain‐derived extracellular vesicle lipids in Alzheimer's disease. J Extracell Vesicles. 2021;10(7):e12089. doi:10.1002/jev2.12089 34012516 10.1002/jev2.12089 PMC8111496

[55] Poitelon Y , Kopec AM , Belin S . Myelin fat facts: an overview of lipids and fatty acid metabolism. Cells. 2020;9(4):812. doi:10.3390/cells9040812 32230947 10.3390/cells9040812 PMC7226731

[56] Kumar M , Wu Y , Knapp J , et al. Triglycerides are an important fuel reserve for synapse function in the brain. Nat Metab. 2025;7(7):1392‐1403. doi:10.1038/s42255‐025‐01321‐x 40595405 10.1038/s42255-025-01321-x PMC12286841

[57] Pryde SE , Duncan SH , Hold GL , Stewart CS , Flint HJ . The microbiology of butyrate formation in the human colon. FEMS Microbiol Lett. 2002;217(2):133‐139. doi:10.1111/j.1574‐6968.2002.tb11467.x 12480096 10.1111/j.1574-6968.2002.tb11467.x

[58] Wei H , Yu C , Zhang C , et al. Butyrate ameliorates chronic alcoholic central nervous damage by suppressing microglia‐mediated neuroinflammation and modulating the microbiome‐gut‐brain axis. Biomed Pharmacother. 2023;160:114308. doi:10.1016/j.biopha.2023.114308 36709599 10.1016/j.biopha.2023.114308

[59] Jaworska J , Zalewska T , Sypecka J , Ziemka‐Nalecz M . Effect of the hdac inhibitor, sodium butyrate, on neurogenesis in a rat model of neonatal hypoxia‐ischemia: potential mechanism of action. Mol Neurobiol. 2019;56(9):6341‐6370. doi:10.1007/s12035‐019‐1518‐1 30767185 10.1007/s12035-019-1518-1 PMC6682584

[60] Senarath R , Oikari LE , Bharadwaj P , Jayasena V , Martins RN , Fernando W . The Therapeutic potential of butyrate and lauric acid in modulating glial and neuronal activity in Alzheimer's disease. Nutrients. 2025;17(14):2286. doi:10.3390/nu17142286 40732911 10.3390/nu17142286 PMC12298293

[61] Chinnappa K , Ballorin F , Francis F . Fundamental neurochemistry review: sphingolipids and ceramides in brain development. J Neurochem. 2025;169(10):e70262. doi:10.1111/jnc.70262 41121737 10.1111/jnc.70262 PMC12541296

[62] Gu H , Territo PR , Persohn SA , et al. Evaluation of chronic lead effects in the blood brain barrier system by DCE‐CT. J Trace Elem Med Biol. 2020;62:126648. doi:10.1016/j.jtemb.2020.126648 32980769 10.1016/j.jtemb.2020.126648 PMC7655551

[63] Mielke MM , Haughey NJ , Han D , et al. The association between plasma ceramides and sphingomyelins and risk of alzheimer's disease differs by sex and APOE in the baltimore longitudinal study of aging. J Alzheimers Dis. 2017;60(3):819‐828. doi:10.3233/JAD‐160925 28035934 10.3233/JAD-160925 PMC5493514

[64] Griffin JWD , Bradshaw PC . Amino acid catabolism in Alzheimer's disease brain: friend or foe?. Oxid Med Cell Longev. 2017;2017:5472792. doi:10.1155/2017/5472792 28261376 10.1155/2017/5472792 PMC5316456

[65] Qi S , Fu J , Li Y , et al. Electrochemical response mechanism of DNA damaged cells: dNA damage repair and purine metabolism activation. Bioelectrochemistry. 2025;161:108832. doi:10.1016/j.bioelechem.2024.108832 39395363 10.1016/j.bioelechem.2024.108832

[66] Morgan RK , Tapaswi A , Polemi KM , et al. Environmentally relevant lead exposure impacts gene expression in SH‐SY5Y cells throughout neuronal differentiation. Toxicol Sci. 2025;207(2):435‐448. doi:10.1093/toxsci/kfaf072 40397994 10.1093/toxsci/kfaf072 PMC12469195

[67] Coppedè F , Migliore L . DNA damage and repair in Alzheimer's disease. Curr Alzheimer Res. 2009;6(1):36‐47. doi:10.2174/156720509787313970 19199873 10.2174/156720509787313970

[68] Hedlich‐Dwyer J , Allard JS , Mulgrave VE , Kisby GE , Raber J , Gassman NR . Novel techniques for mapping dna damage and repair in the brain. Int J Mol Sci. 2024;25(13):7021. doi:10.3390/ijms25137021 39000135 10.3390/ijms25137021 PMC11241736

[69] Nishioka K , Ikezaki M , Iwahashi N , et al. Amyloid‐β fibrils accumulated in preeclamptic placentas suppress cytotrophoblast syncytialization. Life Sci Alliance. 2026;9(4):e202503453. doi:10.26508/lsa.202503453 41558820 10.26508/lsa.202503453 PMC12819053

[70] Fields JA , Garovic VD , Mielke MM , et al. Preeclampsia and cognitive impairment later in life. Am J Obstet Gynecol. 2017;217(1):74.e1‐74.e11. doi:10.1016/j.ajog.2017.03.008 10.1016/j.ajog.2017.03.008 PMC5615406 28322777

[71] Zhong Z , Yang Q , Li C , Chen X , Zhou F . A global perspective of correlation between maternal blood lead levels and risks of preeclampsia: an updated systematic review and meta‐analysis. Front Public Health. 2022;10:1072052. doi:10.3389/fpubh.2022.1072052 36620238 10.3389/fpubh.2022.1072052 PMC9816335

[72] Gupta S , Agarwal A , Sharma RK . The role of placental oxidative stress and lipid peroxidation in preeclampsia. Obstet Gynecol Surv. 2005;60(12):807‐816. doi:10.1097/01.ogx.0000193879.79268.59 16359563 10.1097/01.ogx.0000193879.79268.59

[73] Enkhmaa D , Wall D , Mehta PK , et al. Preeclampsia and vascular function: a window to future cardiovascular disease risk. J Womens Health (Larchmt). 2016;25(3):284‐291. doi:10.1089/jwh.2015.5414 26779584 10.1089/jwh.2015.5414 PMC4790201

[74] Endres D , Tebartz van Elst L , Feige B , et al. On the effect of sex on prefrontal and cerebellar neurometabolites in healthy adults: an MRS study. Front Hum Neurosci. 2016;10:367. doi:10.3389/fnhum.2016.00367 27531975 10.3389/fnhum.2016.00367 PMC4969301

[75] Martin B , Chadwick W , Janssens J , et al. GIT2 Acts as a systems‐level coordinator of neurometabolic activity and pathophysiological aging. Front Endocrinol (Lausanne). 2015;6:191. doi:10.3389/fendo.2015.00191 26834700 10.3389/fendo.2015.00191 PMC4716144

[76] Bresilla D , Habisch H , Pritišanac I , et al. The sex‐specific metabolic signature of C57BL/6NRj mice during aging. Sci Rep. 2022;12(1):21050. doi:10.1038/s41598‐022‐25396‐8 36473898 10.1038/s41598-022-25396-8 PMC9726821

[77] Vints WAJ , Kušleikienė S , Sheoran S , et al. Body fat and components of sarcopenia relate to inflammation, brain volume, and neurometabolism in older adults. Neurobiol Aging. 2023;127:1‐11. doi:10.1016/j.neurobiolaging.2023.02.011 37004309 10.1016/j.neurobiolaging.2023.02.011

