# Supplementary material for: Perinatal lead (Pb) exposure and alterations to amyloid beta and metabolic profiles in the brain
Source: Alzheimers Dement. 2026 Aug 30;22(9):e71791. doi: 10.1002/alz.71791 (PMC13526643; doi:10.1002/alz.71791)
Supplement: Supplementary file 2 [file ALZ-22-e71791-s001.docx]

Figure S2: High magnification representative images of beta amyloid immunostained sample AO45A (A,C,E), sample AO30B (B,D,F), a positive control brain section from a murine Alzheimer’s disease model (G), and a negative control (H). Sample AO45A showed diffuse moderate to high intensity staining in grey matter (A) and sample AO30B showed diffuse low to moderate intensity staining in the same areas (B). Both samples showed highest intensity staining in vascular walls (arrows, A, B). Digital overlays of positive (red) and negative (blue) cells are shown in C, D and digital overlays of the highest intensity pixel areas (arrows) are shown in E,F. The positive control (G) shows numerous extracellular beta amyloid plaques (arrowheads) and also beta amyloid staining in vascular walls (arrow). Negative control shows no staining (H). Bars 50 µm.
