# Supplementary material for: Perinatal lead (Pb) exposure and alterations to amyloid beta and metabolic profiles in the brain
Source: Alzheimers Dement. 2026 Aug 30;22(9):e71791. doi: 10.1002/alz.71791 (PMC13526643; doi:10.1002/alz.71791)
Supplement: Supplementary file 4 [file ALZ-22-e71791-s004.docx]

Figure S1: Representative images from samples AO28J (A), AO45G (B), and AO41H (C) showing thalamic mineralization (A,B) and cerebellar artifactual vacuolation (C). See narrative for discussion. Insets in A, B show low power overview of location of thalamic mineralization. H&E. bars 200 µm (A,B), 300 µm (C).
