# Supplementary material for: Perinatal lead (Pb) exposure and alterations to amyloid beta and metabolic profiles in the brain
Source: Alzheimers Dement. 2026 Aug 30;22(9):e71791. doi: 10.1002/alz.71791 (PMC13526643; doi:10.1002/alz.71791)
Supplement: Supplementary file 5 [file ALZ-22-e71791-s007.docx]

| Sample ID | Submission ID | Lvl2 (slide1) | Lvl3 | Lvl5 |
| --- | --- | --- | --- | --- |
|  |  |  | (slide2) | (slide3) |
| AO27A | 23.3231 | * | * | * |
| AO27G | 23.3231 | * | * | * |
| AO29B | 23.3231 | * | * | * |
| AO29F | 23.3241 | * | * | * |
| AO30B | 23.3241 | * | * | * |
| AO31A | 23.3241 | * | * | * |
| AO32A | 23.3241 | * | * | * |
| AO28C | 23.3241 | * | * | * |
| AO28J | 23.3241 | * | Mineralization, focal, thalamus | * |
| AO40A | 23.3241 | * | Mineralization, focal, thalamus | * |
| AO40G | 23.3241 | * | * | * |
| AO41C | 23.3241 | * | * | * |
| AO41H | 23.3241 | * | * | * |
| AO44B | 23.3241 | * | Mineralization, focal, thalamus | * |
| AO44F | 23.3241 | * | * | * |
| AO45A | 23.3241 | * | * | * |
| AO45G | 23.3241 | * | Mineralization, focal, thalamus | * |
| AO39B | 23.3241 | * | Mineralization, focal, thalamus | * |
| AO29E | 24.83 | * | Mineralization, focal, thalamus | * |
| AO49A | 24.83 | * | * | * |
| AO50C | 24.83 | * | * | * |
| AO50D | 24.83 | * | Mineralization, focal, thalamus | * |
| AO51B | 24.83 | * | * | * |
| AO52A | 24.83 | * | * | Heterotopia, focal, minimal, molecular layer, cerebellum |
| AO52F | 24.83 | * | * | * |
| AO53C | 24.83 | * | * | * |
| AO53I | 24.83 | * | * | * |
| AO60C | 24.172 | * | * | * |
| AO63D | 24.238 | * | * | * |
| PB001D | 24.172 | * | * | * |
| PB002E | 24.172 | * | * | * |
| PB003D | 24.172 | * | * | * |
| PB005D | 24.172 | * | * | * |
| PB005I | 24.172 | * | * | * |
| PB006E | 24.172 | * | * | * |
| PB008F | 24.172 | * | * | * |
| PB011D | 24.172 | * | Mineralization, focal, thalamus | * |
| PB012D | 24.172 | * |  | * |
| PB012I | 24.172 | * | Lateral ventricle dilation, moderate | * |
| PB014G | 24.238 | * | * | * |
| PB015G | 24.238 | * | * | * |

Supplementary Table 2: Summary of H&E and LFB data and pathology interpretation. *no findings. Assessed descriptively from HE, LFB stained slides per standard criteria and nomenclature. Section levels correspond to standard guidelines.
