# Supplementary material for: Perinatal lead (Pb) exposure and alterations to amyloid beta and metabolic profiles in the brain
Source: Alzheimers Dement. 2026 Aug 30;22(9):e71791. doi: 10.1002/alz.71791 (PMC13526643; doi:10.1002/alz.71791)
Supplement: Supplementary file 6 [file ALZ-22-e71791-s009.docx]

**Section 1: Histopathological Methods**

Samples were submitted as mouse brains hemi-sectioned sagittally on midline and immersed in 10% neutral buffered formalin. Brains were further trimmed in the coronal plane post-fixation into ~4 mm thickness standardized section levels allowing an overview of cerebral cortex, anterior commissure, basal ganglia, corpus callosum, thalamic nuclei, hippocampus, cerebral peduncles, midbrain, pons, cerebellum, and cerebellar pyramids (i.e. section levels 2, 3, 5 in Society for Toxicologic Pathology best practices guidelines for sampling the rodent brain)6. These sections were cassetted and processed to paraffin on an automated histology processor (Tissue Tek VIP, Sakura, Torrance, CA). Slides were embedded and sectioned at 4 µm thickness onto glass slides for staining with hematoxylin and eosin or Luxol Fast blue by routine histologic methods. Two to three sections were placed per slide. Slides were evaluated by a board-certified veterinary pathologist using a BX45 Olympus light microscope. Slides were scanned on a digital slide scanner (Leica Aperio AT2, Leica Biosystems, Deer Park, IL) at a resolution of 0.50 µm/pixel (20x objective equivalent). Representative images were taken from the digital slides using manufacturer-provided software (Leica Aperio ImageScope v 12.4.6, Leica Biosystems). Composite images were assembled in Adobe Photoshop CC (v 19.0, Adobe Systems Inc). Photo processing was confined to global adjustment of image size, white balance, brightness, or contrast that did not materially alter the interpretation of the image.

**Histology and immunohistochemistry**

Unstained sections were cut from previously prepared formalin-fixed paraffin-embedded murine brain samples from a previous lead exposure experiment. Sections were cut on a rotary microtome at 4 µm thickness and mounted on glass slides. After deparaffinization, slides underwent heat-induced epitope retrieval (HIER) in a pressure chamber (Decloaking Chamber, Biocare Medical, Pacheco, CA). Immunohistochemical staining was performed on an automated stainer (ONCORE Pro X, Biocare Medical) and included blocking of endogenous peroxidases, primary antibody incubation, and polymer-based detection. The primary antibody (ab201060, Abcam, Cambridge, MA) was a rabbit monoclonal (clone mOC64) targeting monomeric, oligomeric, and fibrillar forms of beta amyloid 1-42 peptide. An archival brain tissue sample from a murine Alzheimer’s disease model with histologically evident beta amyloid plaques was used for initial antibody titration and as a positive control. Conditions and reagents used in primary antibody incubation and detection are given below. Diaminobenzidine (DAB) was applied for 5 minutes as the chromogen (IPK5010G80, Biocare Medical). Following immunostaining, samples were counterstained with hematoxylin for 5 minutes, dehydrated and cleared through graded alcohols and xylene, and cover-slipped. Negative reagent controls followed the same procedure with naïve serum (Universal Negative, IP498G20, Biocare) in place of primary antibody.

**Analysis**

Descriptive and quantitative analysis was performed by a board-certified veterinary pathologist blinded to the experimental groups. Histology slides were digitized at a resolution of 0.27 µm2/pixel on a digital slide scanner (Slideview VS200, Evident Scientific [Olympus Scientific Solutions], Tokyo, Japan). Digital slide files were imported into an open-source image analysis program (QuPath, v0.6.0, available at https://qupath.github.io/).1

Image analysis was performed on a workstation (Dell Precision 3680; Microsoft Windows 11 OS) equipped with a 12th generation Intel Core processor (i7-14700K, 33 MB cache, 20 cores, 28 threads, up to 5.6 GHz Turbo) with 128 GB of installed memory and an additional 8GB installed GPU (NVIDIA, GeForce RTX 4060). Representative image overlays were generated within QuPath and exported as Tiff files.

Intensity of DAB staining was first assessed across the entire tissue area using a pixel classifier for tissue detection and algorithms for stain intensity assessment within QuPath. Analysis parameters are given in Table 3. Color deconvolution was first performed by adjusting RGB stain parameters based on small annotations representing single-stained areas for hematoxylin and DAB. The entire brain sections were automatically selected using a pixel classifier thresholded on the basis of the average intensity across all color channels. Annotations were reviewed by the pathologist and manually exited to exclude artifacts (folds, etc..) and include any true tissue regions erroneously omitted by the classifier. Average and standard deviation for DAB intensity were measured across the whole section at the specified resolution (tile size).

Cells were segmented within the tissue annotation using a previously trained cell segmentation model (InstanSeg) available within QuPath as an extension.2 Because axonal and dendritic projections of neuronal cells are not definable at the resolution of light microscopy, only nuclei and cell bodies were identifiable by segmentation. Cell bodies positive for DAB were identified using a cell classifier plugin within QuPath. Thresholds for determining positive cells were defined empirically by comparison to the negative control, non-staining areas, and lightly staining areas. Thresholds were applied equally across all tissue sections. Analysis parameters are given in Table 4.

High intensity staining seen in capillaries and foci of mineralization were identified using a pixel classifier within QuPath. The threshold defining high intensity staining was determined empirically by visual assessment of the digital overlays and was applied equally across all tissue sections. Analysis parameters are given in Table 5.

Representative field-of-view images were taken from the digital slide files as Tiff files and composite panels were assembled in Adobe Photoshop CC (v 19.0, Adobe Systems Inc). Photo processing was confined to global adjustment of image size, white balance, brightness, or contrast that did not materially alter the interpretation of the image.

**Section 2: Metabolon Sample Processing and Methods**

**Sample Accessioning**: Following receipt, samples were inventoried and immediately stored at -80°C. Each sample received was accessioned into the Metabolon LIMS system and was assigned by the LIMS a unique identifier that was associated with the original source identifier only. This identifier was used to track all sample handling, tasks, results, etc. The samples (and all derived aliquots) were tracked by the LIMS system. All portions of any sample were automatically assigned their own unique identifiers by the LIMS when a new task was created; the relationship of these samples was also tracked. All samples were maintained at -80°C until processed.

**Sample Preparation**: Samples were prepared using the automated MicroLab STAR® system from Hamilton Company. Several recovery standards were added prior to the first step in the extraction process for QC purposes. To remove protein, dissociate small molecules bound to protein or trapped in the precipitated protein matrix, and to recover chemically diverse metabolites, proteins were precipitated with methanol under vigorous shaking for 2 min (Glen Mills GenoGrinder 2000) followed by centrifugation. The resulting extract was divided into multiple fractions: two for analysis by two separate reverse phase (RP)/UPLC-MS/MS methods with positive ion mode electrospray ionization (ESI), one for analysis by RP/UPLC-MS/MS with negative ion mode ESI, one for analysis by HILIC/UPLC-MS/MS with negative ion mode ESI, while the remaining fractions were reserved for backup. Samples were placed briefly on a TurboVap® (Zymark) to remove the organic solvent. The sample extracts were stored overnight under nitrogen before preparation for analysis.

**QA/QC**: Several types of controls were analyzed in concert with the experimental samples: a pooled matrix sample generated by taking a small volume of each experimental sample (or alternatively, use of a pool of well-characterized human plasma) served as a technical replicate throughout the data set; extracted water samples served as process blanks; and a cocktail of QC standards that were carefully chosen not to interfere with the measurement of endogenous compounds were spiked into every analyzed sample, allowed instrument performance monitoring and aided chromatographic alignment. Instrument variability was determined by calculating the median relative standard deviation (RSD) for the standards that were added to each sample prior to injection into the mass spectrometers. Overall process variability was determined by calculating the median RSD for all endogenous metabolites (i.e., non-instrument standards) present in 100% of the pooled matrix samples. Experimental samples were randomized across the platform run with QC samples spaced evenly among the injections.

Ultrahigh Performance Liquid Chromatography-Tandem Mass Spectroscopy (UPLC-MS/MS): All methods utilized a Waters ACQUITY ultra-performance liquid chromatography (UPLC) and a Thermo Scientific Q-Exactive high resolution/accurate mass spectrometer interfaced with a heated electrospray ionization (HESI-II) source and Orbitrap mass analyzer operated at 35,000 mass resolution (PMID: 32445384). The dried sample extract were then reconstituted in solvents compatible to each of the four methods. Each reconstitution solvent contained a series of standards at fixed concentrations to ensure injection and chromatographic consistency. One aliquot was analyzed using acidic positive ion conditions, chromatographically optimized for more hydrophilic compounds (PosEarly). In this method, the extract was gradient eluted from a C18 column (Waters UPLC BEH C18-2.1x100 mm, 1.7 µm) using water and methanol, containing 0.05% perfluoropentanoic acid (PFPA) and 0.1% formic acid (FA). Another aliquot was also analyzed using acidic positive ion conditions; however it was chromatographically optimized for more hydrophobic compounds (PosLate). In this method, the extract was gradient eluted from the same aforementioned C18 column using methanol, acetonitrile, water, 0.05% PFPA and 0.01% FA and was operated at an overall higher organic content. Another aliquot was analyzed using basic negative ion optimized conditions using a separate dedicated C18 column (Neg). The basic extracts were gradient eluted from the column using methanol and water, however with 6.5mM Ammonium Bicarbonate at pH 8. The fourth aliquot was analyzed via negative ionization following elution from a HILIC column (Waters UPLC BEH Amide 2.1x150 mm, 1.7 µm) using a gradient consisting of water and acetonitrile with 10mM Ammonium Formate, pH 10.8 (HILIC). The MS analysis alternated between MS and data-dependent MSn scans using dynamic exclusion. The scan range varied slightly between methods but covered 70-1000 m/z. Raw data files are archived and extracted as described below.

**Bioinformatics**: The informatics system consisted of four major components, the Laboratory Information Management System (LIMS), the data extraction and peak-identification software, data processing tools for QC and compound identification, and a collection of information interpretation and visualization tools for use by data analysts. The hardware and software foundations for these informatics components were the LAN backbone, and a database server running Oracle 10.2.0.1 Enterprise Edition.

**LIMS**: The purpose of the Metabolon LIMS system was to enable fully auditable laboratory automation through a secure, easy to use, and highly specialized system. The scope of the Metabolon LIMS system encompasses sample accessioning, sample preparation and instrumental analysis and reporting and advanced data analysis. All of the subsequent software systems are grounded in the LIMS data structures. It has been modified to leverage and interface with the in-house information extraction and data visualization systems, as well as third party instrumentation and data analysis software.

**Data Extraction and Compound Identification**: Raw data was extracted, peak-identified and QC processed using a combination of Metabolon developed software services (applications). Each of these services perform a specific task independently, and they communicate/coordinate with each other using industry-standard protocols. Compounds were identified by comparison to library entries of purified standards or recurrent unknown entities. Metabolon maintains a library based on authenticated standards that contains the retention time/index (RI), mass to charge ratio (m/z), and fragmentation data on all molecules present in the library. Furthermore, biochemical identifications are based on three criteria: retention index within a narrow RI window of the proposed identification, accurate mass match to the library +/- 10 ppm, and the MS/MS forward and reverse scores between the experimental data and authentic standards. The MS/MS scores are based on a comparison of the ions present in the experimental spectrum to the ions present in the library spectrum. While there may be similarities between molecules based on one of these factors, the use of all three data points is utilized to distinguish and differentiate biochemicals. More than 5,400 commercially available purified or in-house synthesized standard compounds have been acquired and analyzed on all platforms for determination of their analytical characteristics. An additional 7000 mass spectral entries have been created for structurally unnamed biochemicals, which have been identified by virtue of their recurrent nature (both chromatographic and mass spectral). These compounds have the potential to be identified by future acquisition of a matching purified standard or by classical structural analysis. Metabolon continuously adds biologically relevant compounds to its chemical library to further enhance its level of Tier 1 metabolite identifications.

**Compound Quality Control**: A variety of curation procedures were carried out to ensure that a high-quality data set was made available for statistical analysis and data interpretation. The QC and curation processes were designed to ensure accurate and consistent identification of true chemical entities, and to remove or correct those representing system artifacts, mis-assignments, mis-integration and background noise. Metabolon data analysts use proprietary visualization and interpretation software to confirm the consistency of peak identification and integration among the various samples.

**Metabolite Quantification and Data Normalization**: Peaks were quantified using area-under-the-curve. For studies spanning multiple days, a data normalization step was performed to correct variation resulting from instrument inter-day tuning differences. Essentially, each compound was corrected in run-day blocks by registering the medians to equal one (1.00) and normalizing each data point proportionately (termed the “block correction”, Figure 2). For studies that did not require more than one day of analysis, no normalization is necessary, other than for purposes of data visualization. In certain instances, biochemical data may have been normalized to an additional factor (e.g., cell counts, total protein as determined by Bradford assay, osmolality, etc.) to account for differences in metabolite levels due to differences in the amount of material present in each sample.

**Statistical Calculations**: For many studies, two types of statistical analysis are usually performed: (1) significance tests and (2) classification analysis. Standard statistical analyses are performed in ArrayStudio/Jupyter Notebook on log transformed data. For those analyses not standard in ArrayStudio/Jupyter Notebook, the programs R (http://cran.r-project.org/) or JMP are used. Below are examples of frequently employed significance tests and classification methods followed by a discussion of p- and q-value significance thresholds.

**Two-way ANOVA**: ANOVA stands for analysis of variance. For ANOVA, it is assumed that all populations variances are equal. For a two-way ANOVA, three statistical tests are typically performed: the main effect of each factor and the interaction. Suppose we have two factors A and B, where A represents the genotype and B represents the diet in a mouse study. Suppose each of these factors has two levels (A: wild type, knock out; B: standard diet, high fat diet). For this example, there are 4 combinations (“treatments”): A1B1, A1B2, A2B1, A2B2. The overall ANOVA F-test gives the p-value for testing whether all four of these means are equal or whether at least one pair is different. However, we are also interested in the effect of the genotype and diet. A main effect is a contrast that tests the means of one factor across the levels of the other factor. Hence the A main effect compares (mean(A1B1) + mean(A1B2))/2 vs. (mean(A2B1) + mean(A2B2))/2, and the B-main effect compares (mean(A1B1) + mean(A2B1))/2 vs. (mean(A1B2) + mean(A2B2))/2. The interaction is a contrast that tests whether the mean difference for one factor depends on the level of the other factor, which is (mean(A1B2) + mean(A2B1))/2 vs. (mean(A1B1) + mean(A2B2))/2. If the interaction is not significant, then the mean difference in one factor does not depend on the level of the other factor, thus, the main effects are the main tests of interest.

**p-values**: For statistical significance testing, p-values are given. The lower the p-value, the more evidence we have that the null hypothesis (typically that two population means are equal) is not true. If “statistical significance” is declared for p-values less than 0.05, then 5% of the time we incorrectly conclude the means are different, when actually they are the same.The p-value is the probability that the test statistic is at least as extreme as observed in this experiment given that the null hypothesis is true. Hence, the more extreme the statistic, the lower the p-value and the more evidence the data gives against the null hypothesis.

**q-values**: The level of 0.05 is the false positive rate when there is one test. However, for a large number of tests we need to account for false positives. There are different methods to correct for multiple testing. The oldest methods are family-wise error rate adjustments (Bonferroni, Tukey, etc.), but these tend to be extremely conservative for a very large number of tests. With gene arrays, using the False Discovery Rate (FDR) is more common. The family-wise error rate adjustments give one a high degree of confidence that there are zero false discoveries. However, with FDR methods, one can allow for a small number of false discoveries. The FDR for a given set of compounds can be estimated using the q-value.
