# Supplementary material for: Perinatal lead (Pb) exposure and alterations to amyloid beta and metabolic profiles in the brain
Source: Alzheimers Dement. 2026 Aug 30;22(9):e71791. doi: 10.1002/alz.71791 (PMC13526643; doi:10.1002/alz.71791)
Supplement: Supplementary file 7 [file ALZ-22-e71791-s011.docx]

| Sample ID | Mean DAB intensity across all tissue | Standard deviation intensity across all tissue | Number of positive cells per tissue area (mm^2^) (DAB threshold 0.4) | Percent area of highest intensity foci (DAB threshold 1.0) |
| --- | --- | --- | --- | --- |
|  |  |  |  |  |
| AO27A | 0.2 | 0.11 | 131.88 | 0.09 |
| AO27G | 0.16 | 0.07 | 40.6 | 0.01 |
| AO28C | 0.2 | 0.09 | 86.33 | 0.03 |
| AO28J | 0.3 | 0.12 | 367.48 | 0.07 |
| AO29B | 0.17 | 0.09 | 79.54 | 0.05 |
| AO29E | 0.29 | 0.12 | 374.58 | 0.1 |
| AO29F | 0.18 | 0.08 | 61.89 | 0.05 |
| AO30B | 0.15 | 0.09 | 89.91 | 0.07 |
| AO31A | 0.19 | 0.1 | 91 | 0.02 |
| AO32A | 0.22 | 0.09 | 117.14 | 0.02 |
| AO39B | 0.26 | 0.11 | 184.36 | 0.05 |
| AO40A | 0.21 | 0.14 | 260.22 | 0.12 |
| AO40G | 0.28 | 0.14 | 468.05 | 0.14 |
| AO41C | 0.21 | 0.11 | 148.53 | 0.02 |
| AO41H | 0.23 | 0.11 | 212.64 | 0.07 |
| AO44B | 0.28 | 0.11 | 260.42 | 0.08 |
| AO44F | 0.33 | 0.15 | 775.98 | 0.18 |
| AO45A | 0.28 | 0.13 | 457.53 | 0.1 |
| AO45G | 0.28 | 0.11 | 290.91 | 0.06 |
| AO49A | 0.24 | 0.14 | 281.57 | 0.27 |
| AO50C | 0.24 | 0.12 | 225.59 | 0.06 |
| AO50D | 0.27 | 0.11 | 271.01 | 0.09 |
| AO51B | 0.2 | 0.1 | 119.88 | 0.05 |
| AO52A | 0.3 | 0.14 | 430.92 | 0.24 |
| AO52F | 0.25 | 0.12 | 314.95 | 0.06 |
| AO53C | 0.3 | 0.11 | 242.67 | 0.06 |
| AO53I | 0.3 | 0.12 | 703.56 | 0.07 |
| AO60C | 0.26 | 0.12 | 394.71 | 0.06 |
| AO63D | 0.29 | 0.12 | 876.86 | 0.06 |
| PB001D | 0.3 | 0.13 | 420.65 | 0.11 |
| PB002E | 0.26 | 0.12 | 286.11 | 0.05 |
| PB003D | 0.32 | 0.14 | 571.91 | 0.11 |
| PB005D | 0.25 | 0.11 | 257.92 | 0.07 |
| PB005I | 0.3 | 0.12 | 422.3 | 0.08 |
| PB006E | 0.28 | 0.12 | 369.87 | 0.05 |
| PB008F | 0.26 | 0.12 | 312.2 | 0.1 |
| PB011D | 0.24 | 0.11 | 172.79 | 0.05 |
| PB012D | 0.27 | 0.11 | 282.02 | 0.09 |
| PB012I | 0.23 | 0.09 | 118.9 | 0.02 |
| PB014G | 0.28 | 0.13 | 501.37 | 0.08 |
| PB015G | 0.24 | 0.11 | 221.46 | 0.07 |

Supplementary Table 3: Summary of amyloid-B staining immunohistopathology.
