# Supplementary material for: Perinatal lead (Pb) exposure and alterations to amyloid beta and metabolic profiles in the brain
Source: Alzheimers Dement. 2026 Aug 30;22(9):e71791. doi: 10.1002/alz.71791 (PMC13526643; doi:10.1002/alz.71791)
Supplement: Supplementary file 8 [file ALZ-22-e71791-s002.docx]

Supplementary Table 1: Sample size summary for metabolomics untargeted profiling.
